# The 14-3-3 Protein BdGF14a Increases the Transcriptional Regulation Activity of BdbZIP62 to Confer Drought and Salt Resistance in Tobacco

**DOI:** 10.3390/plants13020245

**Published:** 2024-01-15

**Authors:** Yang Zhang, Yuan He, Hongyan Zhao, Yan Wang, Chunlai Wu, Yuanzeng Zhao, Hongna Xue, Qidi Zhu, Jinlong Zhang, Xingqi Ou

**Affiliations:** 1 Henan Institute of Science and Technology, School of Agriculture, Xinxiang 453003, China; tstcedufeiyang@163.com (Y.Z.); xuehongna721@163.com (H.X.); zzhhqqdd@163.com (Q.Z.); 2 The Genetic Engineering International Cooperation Base of Chinese Ministry of Science and Technology, The Key Laboratory of Molecular Biophysics of Chinese Ministry of Education, College of Life Science and Technology, Huazhong University of Science & Technology, Wuhan 430074, China; circlecirclehe@163.com (Y.H.); zhaohy@hust.edu.cn (H.Z.); wy875643001wy@163.com (Y.W.); wuchunlai@henau.edu.com (C.W.); 3 Henan Institute of Science and Technology, School of Life Sciences, Xinxiang 453003, China; yuanzengzh@126.com

**Keywords:** drought and salt stresses, BdGF14a, BdbZIP62, ABA, transcriptional regulation activity

## Abstract

*BdGF14a*, a *14-3-3* gene from *Brachypodium distachyon*, induced by salt, H_2_O_2_, and abscisic acid (ABA), improved tolerance to drought and salt in tobacco, with a higher survival rate and longer roots under these stresses. Additionally, physiological index analyses showed that the heterologous expression of *BdGF14a* induced higher expression levels of antioxidant enzymes and their activities, leading to lighter DAB and NBT staining, denoting decreased H_2_O_2_ content. Additionally, the lower MDA content and ion leakage indicated enhanced cell membrane stability. Moreover, exogenous ABA resulted in shorter roots and a lower stomatal aperture in *BdGF14a* transgenic plants. BdGF14a interacted with NtABF2 and regulated the expression of stress-related genes. However, adding an ABA biosynthesis inhibitor suppressed most of these changes. Furthermore, similar salt and drought resistance phenotypes and physiological indicators were characterized in tobacco plants expressing BdbZIP62, an ABRE/ABF that interacts with BdGF14a. And Y1H and LUC assays showed that BdGF14a could enhance the transcription regulation activity of NtABF2 and BdbZIP62, targeting *NtNECD1* by binding to the ABRE *cis*-element. Thus, BdGF14a confers resistance to drought and salinity through interaction with BdbZIP62 and enhances its transcriptional regulation activity via an ABA-mediated signaling pathway. Therefore, this work offers novel target genes for breeding salt- and drought-tolerant plants.

## 1. Introduction

Various environmental stresses, such as drought and high salinity, restrict plant growth and development, especially in staple cereal crops, and they cause agricultural yield loss; under stress, plants utilize a series of signal transduction pathways [1,2]. A group of adaptor proteins called 14-3-3s was recently shown to play an important function in the response to abiotic stresses [3,4]. They are highly conserved intra- and inter-specifically [5], and they are involved in growth and development, light signal transduction, and hormone-based regulation in plants [6,7,8,9,10]. Additionally, several reports have demonstrated the essential functions of 14-3-3s in coping with salt- and drought-induced stresses [4]. Ca^2+^-induced 14-3-3s act as a molecular switch during the response to salt stress [11]. Subjected to high salt, plants maintain ion homeostasis by limiting Na^+^ accumulation in cells [1,12]. Plants mainly respond to a high-salt environment via the SOS signaling pathway, vital in adjusting intracellular ion homeostasis [12]. In *Arabidopsis*, under a low-salt environment, 14-3-3λ and κ bound to SOS2 kinase and suppressed its activity, while under a high-salinity environment, SOS2 dissociated from the complex and participated in the SOS signaling pathway [13]. 14-3-3s bind to the SOS1 C-term, and this might be another regulatory mechanism for coping with salt-induced stress [14]. The regulation of the localization and stability of 14-3-3s is another layer of the salt resistance mechanism in plants [15]. In addition, the negative and positive regulation of MdGRF6 and TaGRF6-A during salt-induced stress, respectively [16,17], indicate the diverse functions of 14-3-3s under salt stress. Additionally, the heterologous expression of *At14-3-3λ* in cotton enhanced the tolerance to drought-induced stress [18]. GRF9 and Hv14-3-3A acted as positive factors under water-induced stress by regulating root or stomatal development [19,20]. GsGF14o induced root hair development and rapid stomatal regulation while coping with drought [21]. ZmGF14-6 improved the resistance to aridity by enhancing the transcription of stress-related genes [22]. OsGF14f positively mediated abscisic acid (ABA)-based responses induced by drought via interaction with OsbZIP23 [23,24], illustrating the positive effect of 14-3-3s in coping with drought-induced stress.

Moreover, several *14-3-3* genes enhance the resistance to simultaneous salt- and drought-induced stresses, such as the one encoding MdGRF11 in apple [25], which interacted with MdARF19-2 and regulated the expression of genes associated with reactive oxygen species (ROS) scavenging to cope with drought-induced stress [26]. OsGF14b plays a vital function in dealing with these stresses through the ABA-dependent signaling pathway or by promoting the stability of its interacting protein OsPLC1 [27,28]. TaGF14b strengthened the resistance to salt- and drought-induced stresses in transgenic tobacco plants by enhancing the ABA content and promoting ABA-based signaling [29].

Although elucidating the functions of 14-3-3s against abiotic stress has progressed, only two *14-3-3* genes from *Brachypodium distachyon* (*B. distachyon*), a model plant of Triticeae crops, have been functionally studied in response to abiotic stress [30]. Bradi3g38640 (BdGF14a) improved drought resistance in *Arabidopsis* [31]. In a previous study by our group, BdGF14d conferred salt resistance by being involved in the antioxidase system; ion transport; and interaction with two ABA-responsive element (ABRE)-binding factors (ABFs) (ABRE/ABF), BdbZIP62 and BdbZIP71 [32]. Until now, only a limited number of reports on the role of 14-3-3s in providing resistance to salt- and drought-induced stresses in *B. distachyon* are available.

In addition, ABA, a cardinal phytohormone, functions in the response of plants to abiotic stress by participating in various processes such as stomatal closure, the inhibition of seed germination, and growth regulation [2,33,34,35]. The ABRE/ABF transcription factor (TF) ABI5, belonging to the group A subfamily of bZIP, which interacts with 14-3-3s, was one of the prominent regulators of the ABA signaling pathway, and it responded to abiotic stresses [21,32,33,36]. Nevertheless, to date, limited evidence has verified the relationship between the resistance to drought- and salt-induced stresses conferred by 14-3-3s and ABRE/ABF-modulated ABA signaling. OsbZIP23, a notable ABRE/ABF TF, interacted with OsGF14f and activated the expression of downstream target genes by binding to their promoters, which, in turn, feedback-regulated ABA accumulation or the formation of an OsbZIP23–OsGF14f complex while coping with water deficiency [23,24]. This is the only study to investigate the mechanism by which the ABRE/ABF–GF14 complex responds to drought and whether the “ABF–GF14f” model plays a role in the response of *B. distachyon* to abiotic stress.

This report identified a *14-3-3* designated *BdGF14a* (Bradi1g11290.1) and an ABRE/ABF TF, *BdbZIP62* (Bradi3g57960.1), from *B. distachyon*. Multiple stress treatments upregulated the transcription of *BdGF14a* and *BdbZIP62*. The overexpression of *BdGF14a* or *BdbZIP62* in tobacco plants improved the resistance to drought and salt and led to hypersensitivity to exogenous ABA. The enhanced resistance-associated phenotype was attributed to improved ROS scavenging abilities and/or the enhanced expressions of stress-associated genes, as well as to the regulation of ABA-related signaling in *BdGF14a*-expressing tobacco plants. BdGF14a interacted with NtABF2/BdbZIP62 and enhanced their transcriptional regulation activity by binding to the ABRE *cis*-element of the promoter of *NtNECD1* to regulate ABA signaling, leading to a response to salt- and drought-induced stresses. This finding revealed for the first time the biological functions of BdGF14a and BdbZIP62 in coping with these stresses, and it also provides fresh evidence affirming the recently developed model of the GF14-bZIP-regulated osmotic response [24]. And these results extend the scope of its application to improving the resistance to salt-induced stress.

## 2. Results

### 2.1. BdGF14a Was Induced by Multifarious Stresses, and the Encoded Protein Localized to the Entire Onion Cell

The cDNA sequence of the *14-3-3* gene *Bradi1g11290.1*, belonging to the non-ε subgroup, was cloned from *B. distachyon*, with an ORF of 783 bp long and encoding a protein 260 amino acids long (GenBank: KU933262.1). A phylogenetic analysis and sequence alignment revealed that BdGF14a had a high sequence homology with all the detected proteins, with a 99% similarity to HvGF14a (GenBank: X62338.1) and a 98% similarity to TaGF14h (GenBank: 8D47B4C37.1) (Figure S1). Owing to the high homology with HvGF14a, the 14-3-3 protein identified was designated as “BdGF14a”. In addition, 14-3-3s were highly conserved, even across plant species.

The expression patterns analyzed using qRT-PCR revealed that *BdGF14a* was detected in all the selected organs with different transcription levels, with the highest being in the leaves (Figure S2A), and the expression was upregulated by NaCl, H_2_O_2_, and ABA (Figure S2B–F). These results suggest that *BdGF14a* was responsive to abiotic stresses and may participate in ABA- and H_2_O_2_-based signaling pathways. A subcellular localization analysis showed that the green fluorescence signal of *pBI121*-*BdGF14a*-*GFP* was detected throughout the epidermal cells of onions (Figure S3), illustrating that BdGF14a was expressed throughout the entire onion cell.

### 2.2. BdGF14a Enhances the Resistance to Drought and Salt in Tobacco

**Figure 1:**
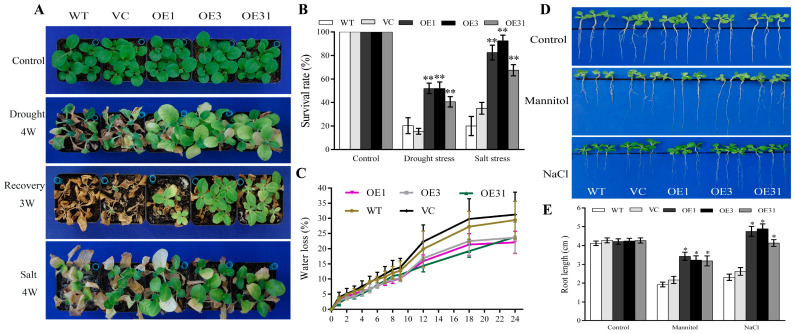
BdGF14a contributed to the tolerance to salt and drought in transgenic tobacco. (**A**) The phenotypic identification of the *BdGF14a* transgenic seedlings before treatment, dehydrated for 6 weeks and re-watered for 3 weeks, or treated with a 500 mM NaCl solution for 4 weeks was performed. (**B**) The survival rates of *BdGF14a*-expressing tobaccos under drought and salt stresses. (**C**) The water loss rates of detached leaves in WT, VC, and *BdGF14a* overexpression tobaccos were ascertained every 2 h. (**D**) Root length assay under different stresses. Two-week-old tobacco seedlings were grown on 1/2 MS medium with or without 150 mM mannitol and 100 mM NaCl for 2 weeks and photographed. (**E**) The root lengths were measured following treatments. Asterisks indicate statistically significant differences (* *p* < 0.05, ** *p* < 0.01; Student’s *t*-test).

The model plant tobacco (*Nicotiana tabacum* L.) was transformed with *CaMV35s*-*BdGF14a* to further ascertain the functions of *BdGF14a* under abiotic stresses. Three stable independent T_3_ transgenic lines with different expression levels of *BdGF14a*, designated as OE1, OE3, and OE31, were selected for stress-related studies. The phenotypes of mature seedlings were first analyzed. For drought treatment, four-week-old tobacco plants were deprived of water. After four weeks of treatment, the transgenic tobacco lines exhibited better growth than most vector control (VC) and wild-type (WT) plants (Figure 1A). Then, following re-watering for three weeks after withholding water for six weeks, certain parts of the transgenic plants recovered to the expected growth status, and the transgenic plants achieved higher survival rates of 40–55% than the controls (16–20%) (Figure 1A,B). Additionally, after four weeks of treatment with 500 mM NaCl, the three *BdGF14a*-expressing lines had more greenish leaves and higher survival rates of 60–85% than the VC and WT plants at 20–38% (Figure 1A,B). These results confirm that BdGF14a strengthened the resistance to drought and salt treatments in the late-stage tobacco seedlings.

As the water content of the leaves is a vital index indicating the abiotic stress tolerance of plants, the water loss rates were calculated in the *BdGF14a*-expressing plants. The results revealed no apparent differences between the tobacco plants tested before 9 h of treatment. However, the leaves of the *BdGF14a*-expressing plants exhibited a notably reduced water loss than those of the controls after 9 h (Figure 1C). The results revealed that BdGF14a could improve the water conservation ability in leaves, thereby improving stress tolerance.

A root length assay was performed using the early-stage seedlings of the *BdGF14a*-expressing tobacco lines to further ascertain their responses to salt and drought conditions. The control and transgenic lines demonstrated a similar growth status, and there were no significant differences in the root lengths on half MS. However, the root lengths were much longer in the *BdGF14a* transgenic lines than in the control lines grown on 1/2 MS supplemented with 100 mM NaCl or 150 mM mannitol (Figure 1D,E). The results indicate that the transgenic seedlings grew better under NaCl and mannitol at the seedling stage, with a phenotype similar to that of adult plants.

### 2.3. BdGF14a Decreases the IL and MDA Contents but Increases the Proline and Sugar Contents and the Tolerance to Oxidative-State-Induced Stress

**Figure 2:**
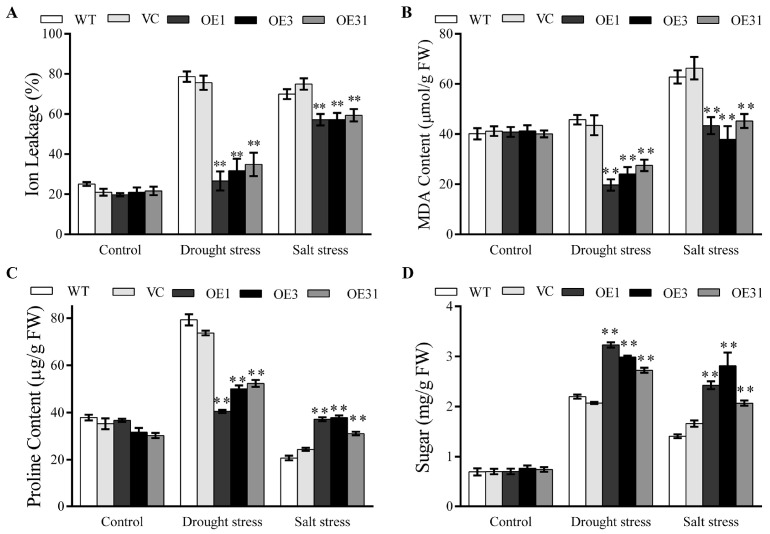
The physiological indices of the control and *BdGF14a* transgenic plants under drought and salt stresses. The ion leakage (**A**) and contents of MDA (**B**), proline (**C**), and sugar (**D**) were measured in *BdGF14a*-expressing plants under normal and stress-induced (drought and salt) treatments. Asterisks indicate statistically significant differences (** *p* < 0.01; Student’s *t*-test).

Physiological indices, including IL, and the contents of MDA, sugar, and proline, indicating tolerance levels in plants, were determined. There were no significant differences in these parameters under normal conditions. However, compared to the controls, lower IL and lower MDA and proline contents but a higher sugar accumulation were observed in the *BdGF14a*-expressing tobaccos under drought (Figure 2). Under salt, lower IL and lower MDA contents but more elevated sugar and proline levels were detected in the *BdGF14a* overexpression tobaccos than the WT and VC plants (Figure 2). The results suggest that the *BdGF14a*-expressing tobaccos suffered lighter membrane oxidative damage and synthesized more stress-related compounds than the WT and VC plants under dry and saline environments.

**Figure 3:**
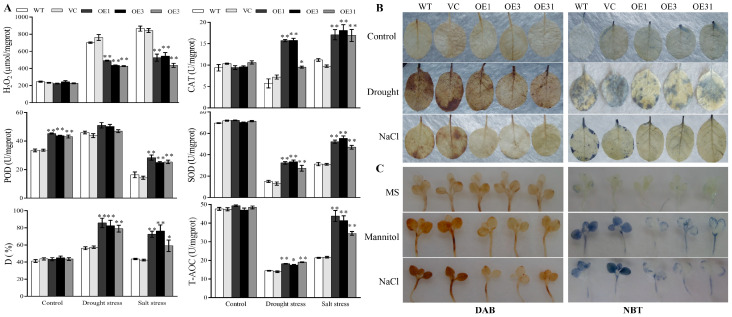
BdGF14a enhances the ROS scavenging abilities and decreases ROS accumulation. (**A**) The ROS-related physiological indices of the control and *BdGF14a*-expressing tobaccos under normal and stress-inducing conditions. The H_2_O_2_ contents, antioxidant enzyme activities (including those of CAT, POD, and SOD), hydroxyl free radical scavenging activity (abbreviated as D), and total antioxidant capacity (T-AOC) were measured. (**B**) DAB and NBT staining in adult plants with large leaves similar to those of four-week-old tobacco grown on sand and dehydrated for 1 week or treated with 500 mM NaCl for 3 weeks. (**C**) DAB and NBT staining of the leaves of 2-week-old seedlings (early stage) grown treated on 1/2 MS medium containing 300 mM mannitol or 200 mM NaCl for 2 days. The photographs were taken after decolorization. Asterisks indicate statistically significant differences (* *p* < 0.05, ** *p* < 0.01; Student’s *t*-test).

ROS could be induced in plant cells by stress, which then causes cellular damage. Therefore, the enzyme activities of the ROS scavenging system were ascertained. Under normal conditions, except for peroxidase (POD), no significant differences were detected between the transgenic tobacco lines and control plants (Figure 3A). Under salt and drought, the *BdGF14a* transgenic tobacco plants demonstrated higher activities of catalase (CAT), POD, and superoxide dismutase (SOD) and a lower H_2_O_2_ content than the controls. In addition, the *BdGF14a* transgenic tobacco plants had a higher relative hydroxyl radical scavenging ability (D) and total antioxidant capacity (T-AOC) under salt and drought conditions (Figure 3A). Correspondingly, staining with 3,3′-diaminobenzidine (DAB) and nitroblue tetrazolium (NBT) indicated that the three *BdGF14a*-expressing tobacco lines were lighter in color than the control plants under stress at the growth stages of young seedlings and adult plants (Figure 3B,C). These results illustrate that *BdGF14a* induced excellent ROS scavenging ability by elevating the CAT, SOD, POD, D, and T-AOC activities and alleviated oxidative damage to enhance salinity and drought resistance.

Then, methyl viologen (MV) was used to further examine the role of *BdGF14a* under oxidative-state-related stress. The leaves of all plants remained green in distilled water. However, the leaves of the *BdGF14a*-expressing tobacco lines were much greener, with a higher chlorophyll accumulation than the WT plants in an MV solution (Figure S4), indicating that *BdGF14a* enhanced the tolerance to stress related to the oxidative state.

### 2.4. BdGF14a Regulates the Expression of Stress-Related Genes under Salt and Drought Stresses

**Figure 4:**
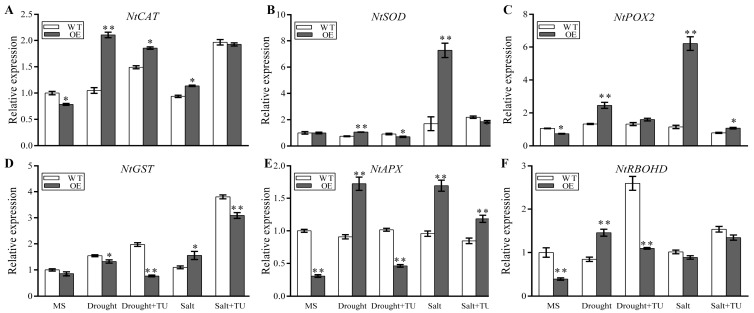
Expression patterns of genes involved in ROS scavenging in *BdGF14a* transgenic tobaccos under drought and salt treatments supplemented with or without 0.1 mM sodium tungstate (Tu) for five days. The expression levels of the ROS-scavenging-related genes, including *NtCAT* (**A**), *NtSOD* (**B**), *NtPOX2* (**C**), *NtGST* (**D**), *NtAPX* (**E**), and *NtRBOHD* (**F**), were analyzed using qRT-PCR. Asterisks indicate statistically significant differences (* *p* < 0.05, ** *p* < 0.01; Student’s *t*-test).

The transcription of most ROS-related genes, *NtCAT*, *NtSOD*, *NtPOX2*, *NtGST* (glutathione S-transferase), *NtAPX* (ascorbate peroxidase), and *NtRBOHD*; ion-channel-encoding genes, *NtSOS1* (plasma membrane Na^+^/H^+^ antiporter), *NtCAX3*/*NtCAX2* (Ca^2+^/H^+^ exchanger), *NtNKT1* and *NtNHX4/NtNHX2* (Na^+^/H^+^ antiporter), and *NtNHX2*; and other stress-related genes, *NtSPSA* (sucrose-phosphate synthase), *NtSUS1* (sucrose synthase), *NtADC1* (arginine decarboxylase), *NtSAMDC* (S-adenosylmethionine decarboxylase), and *NtP5CS1* (11-pyrroline-5-carhoxylate synthetase 1), were higher in the *BdGF14a*-expressing tobaccos than in the control plants under drought and salinity (Figure 4, Figures S5 and S6). The addition of sodium tungstate (Tu), an inhibitor of ABA biosynthesis, suppressed the upregulation of these genes under abiotic stress treatments (Figure 4, Figures S5 and S6). These results demonstrate that *BdGF14a* could regulate the expression of stress-related genes to enhance salt and drought tolerance, and the upregulation of these genes relied on ABA.

### 2.5. BdGF14a Decreases Stomatal Aperture under Abiotic Stresses and Increases ABA Sensitivity in Tobaccos

**Figure 5:**
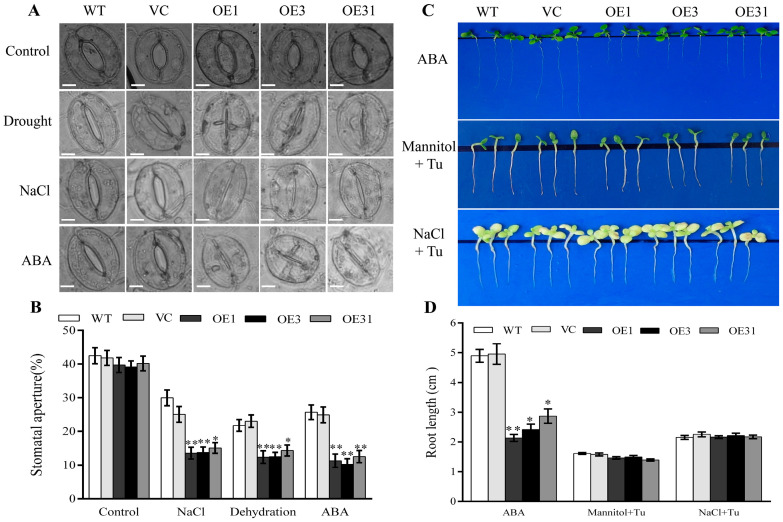
*BdGF14a* modulated stomata movement under salt and drought treatments and increased ABA sensitivity. (**A**) The stomata movement of *BdGF14a* transgenic lines under dehydration, 200 mM NaCl, and 50 μM ABA treatments. A bright-field fluorescence microscope was used to capture the images. Scale bar = 5 μM. (**B**) The stomatal apertures were measured. (**C**) Two-week-old seedlings of controls and T_3_ *BdGF14a*-overexpressing tobacco plants were grown on 1/2 MS medium with 10 μM ABA, 150 mM mannitol/100 mM NaCl + 0.1 mM sodium tungstate (Tu) for two weeks, and then the photographs were taken. (**D**) Root length analysis. Asterisks indicate statistically significant differences (* *p* < 0.05, ** *p* < 0.01; Student’s *t*-test).

As the stoma is an essential gateway for water loss in plants via transpiration, it is considered a vital index of tolerance to stress. Therefore, the width/length ratio of the stoma was measured before and after treatment. All stomata of the pre-treated control and *BdGF14a* transgenic tobaccos were open, and the stoma apertures were similar among these lines. However, after dehydration and treatment with the salt solution, the stomata of the three *BdGF14a*-expressing plants were almost closed with a lower aperture, while those of the VC and WT plants were still open (Figure 5A,B). The results show that the speed of stomatal closure was increased to reduce water loss and improve the tolerance to salt and drought in the *BdGF14a* transgenic tobaccos.

ABA is a critical abiotic stress-responsive phytohormone in plants [37,38] and functions in stomatal closure and growth regulation [34,35,39]. The leaves of the *BdGF14a* transgenic and control plants were exposed to 50 μM ABA to identify whether their guard cells responded to ABA. As shown in Figure 4A, the stomata of the *BdGF14a* overexpression tobaccos were almost closed, while most of the stomata of the WT and VC plants were still open; this also agrees with the statistical results (Figure 5B). These results illustrate that *BdGF14a* took part in ABA-regulated stomatal closing. In addition, the roots of the *BdGF14a* transgenic tobaccos were shorter than those of the control plants grown on a medium with exogenous ABA (Figure 5C), indicating that it enhanced the sensitivity of young seedlings and adult plants to ABA. Interestingly, the addition of Tu suppressed the elongation of the roots under NaCl- and mannitol-induced stresses, and no notable differences were detected between all the tested tobaccos (Figure 5C,D), implying that the tolerance to abiotic stress tolerance conferred by *BdGF14a* was dependent on ABA.

### 2.6. BdGF14a Interacts with NtABF2 and Regulates the Expressions of ABA-Related Genes

**Figure 6:**
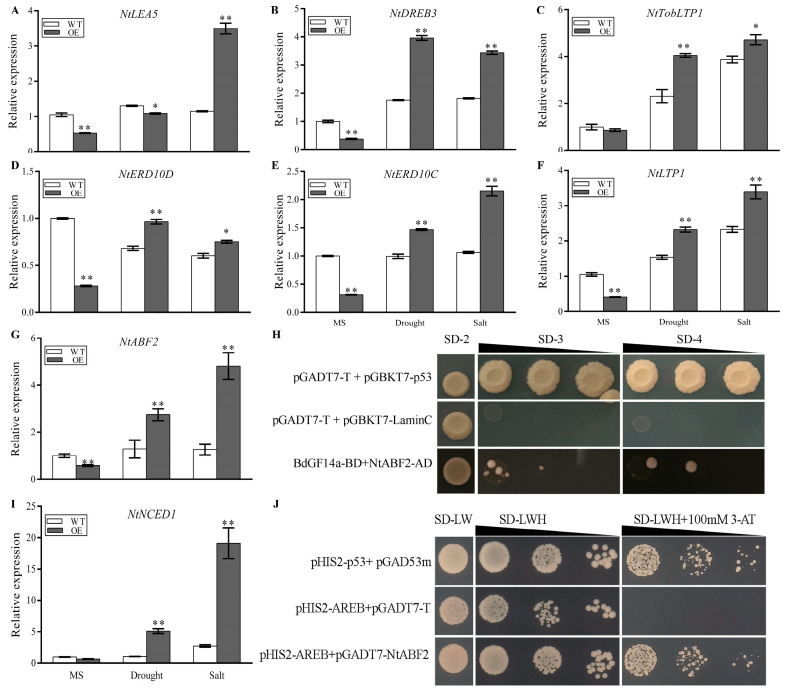
BdGF14a interacted with NtABF2 in the Y2H assay and regulated the expression of ABA-related genes. (**A**) The expression levels of ABA-related genes, including *NtLEA5* (**A**), *NtDREB3* (**B**), *NtTobLTP1* (**C**), *NtERD10D* (**D**), *NtERD10C* (**E**), *NtLTP1* (**F**), and *NtABF2* (**G**), were ascertained using qRT-PCR. (**H**) Y2H assay revealed that BdGF14a interacted with NtABF2. BdGF14a was transferred into the vector pGBKT7 (BD), and NtABF2 was cloned into the vector pGADT7 (AD). They were then co-transformed into the AH109 strain and spotted onto the selective medium SD/-Trp-Leu (SD-2), SD/-Trp-Leu-Ade (SD-3), or SD/-Trp-Leu-His-Ade (SD-4). The co-transformants of pGADT7-*T* and pGBKT7-*LaminC* or pGBKT7-p*53* represented the controls. (**I**) qRT-PCR analysis of *NtNCED1* expression after exposure to abiotic stress. (**J**) Y1H assay indicated that NtABF2 interacted with the ABRE *cis*-element. The recombinant vectors pHIS2-ABRE and pGADT7-NtABF2 were co-transformed into Y187 and plated on the SD-2 selection medium at 30 °C for 2–3 d. Then, they were spotted onto SD-LW, SD-LWH, and SD-LWH + 100 mM 3-AT for another 3 d. The co-transformed pHIS2-ABRE + pGADT7-T and pGAD53m + pHIS2-P53 were used as the negative and positive controls. Asterisks indicate statistically significant differences (* *p* < 0.05, ** *p* < 0.01; Student’s *t*-test).

To better reveal the molecular basis of *BdGF14a*-conferred tolerance to salt and drought, the transcription levels of selected ABA-related genes were analyzed. Under normal conditions, except for *NtNCED1* (9-cis-epoxy carotenoid dioxygenase) and *NtTobLTP1* (lipid transfer protein), the transcription of most of the ABA-related genes was remarkably lower in the *BdGF14a*-expressing lines than in the controls (Figure 6). Under drought and salinity, besides *NtLEA5* (late embryogenesis abundant 6), the transcription of all the other genes, namely, *NtNCED1*, *NtABF2*, *NtTobLTP1*, *NtLPT1*, *NtDREB3* (dehydration-responsive element binding protein), and *NtERD10C/D* (early response to dehydration), in the *BdGF14a*-expressing tobaccos was higher than in the WT plants. The transcript level of *NtLEA5* was higher under salt but lower under drought compared to the WT plants (Figure 6A–G,I).

ABF2 was involved in the abiotic stress response by regulating the transcription of downstream ABA-responsive genes by binding to the ABRE element [23,24,40,41]. BdGF14a was reported to interact with three BdABRE/ABF TFs [32], and it could regulate the expression of *NtNCED1*, a potential target of ABF2 (Figure 6I); thus, yeast two-hybrid (Y2H) and yeast one-hybrid (Y1H) experiments were performed to detect the interaction of NtABF2 with the BdGF14a/ABRE *cis*-element. As expected, NtABF2 interacted with BdGF14a, allowing for cell survival on the SD/-Trp-Leu-Ade (SD-3) and SD/-Trp-Leu-His-Ade (SD-4) selective media (Figure 6H). The co-transformed yeast cells of NtABF2 and the ABRE *cis*-element had a better growth status on the SD/-Trp/-Leu/-His (SD-LWH) + 100 mM 3-amino-1,2,4-triazole (3-AT) selection plates than the empty vector cells (Figure 6J), indicating that BdGF14a interacted with NtABF2, an ABRE/ABF TF, which bound to the ABRE *cis*-element of the target genes and might regulate their expression.

### 2.7. BdbZIP62, Interacted with BdGF14a, Positively Regulates the Response to to Drought and Salt Stresses

BdbZIP62 (Bradi3g57960.1), an orthologous protein of NtABF2 and an essential transcriptional regulator of the ABRE-dependent ABA signaling pathway involved in the abiotic stresses response, was revealed to interact with BdGF14a in a previous study by our group [32]. To determine the functional role of BdbZIP62 in the BdGF14a-dependent response to abiotic stresses, the expression of *BdbZIP62* in different tissues and treatments was first ascertained using qRT-PCR. The differential expression of *BdbZIP62* was detected in all the selected tissues, with the highest levels detected in the roots and leaves (Figure S7A). Additionally, its expression could be induced in the 14-day-old leaves of the *B. distachyon* seedlings treated with PEG, NaCl, H_2_O_2_, and ABA, which peaked at 6 or 12 h of treatment (Figure S7B–F). These observations show that, similar to BdGF14a, BdbZIP62 was responsive to salinity and drought, which might involve ABA- and H_2_O_2_-dependent signaling pathways.

**Figure 7:**
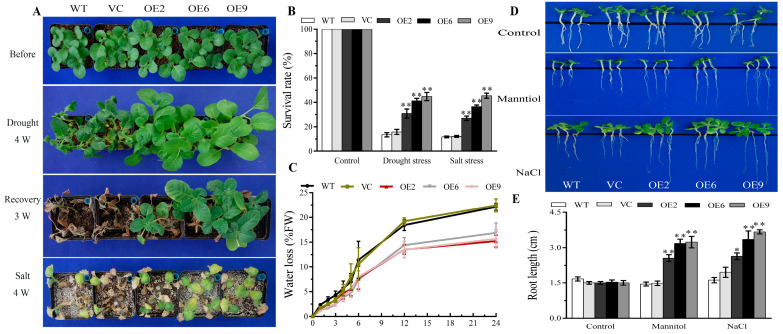
*BdbZIP62*-overexpressing transgenic tobaccos exhibited increased resistance to drought and salt stress. (**A**) The phenotype analysis under drought and salt. (**B**) The survival percentage of plants after abiotic stress treatments. (**C**) The water loss rate analysis. (**D**) The root length assay of two-week-old seedling plants on 1/2 MS medium with or without 150 mM mannitol and 100 mM NaCl for 2 weeks. (**E**) The root lengths were measured after treatments. Asterisks indicate statistically significant differences (* *p* < 0.05, ** *p* < 0.01; Student’s *t*-test).

Three stable and independent T_3_ *BdbZIP62* transgenic lines, designated OE2, 6, and 9, were obtained to characterize the role of BdbZIP62 in coping with abiotic stresses. The phenotypic responses of the *BdbZIP62*-expressing tobacco plants to drought and salt conditions were observed. The results are similar to those of BdGF14a, with the survival rates being 15–31.2% and 14.8–33.2% higher in the *BdbZIP62* overexpression tobaccos than in WT and VC under drought and salt treatments, respectively (Figure 7A,B).

The analysis of the water loss rates showed no apparent variations between the transgenic and control tobaccos before 5 h of treatment. However, the *BdbZIP62* transgenic tobacco lines showed notably lower water loss than the controls after 5 h (Figure 7C). These results revealed that *BdbZIP62* could improve the ability of the leaves to control water loss. Furthermore, the roots of the three *BdbZIP62*-expressing tobacco lines being longer than those of the WT and VC plants on a medium supplemented with mannitol or NaCl (Figure 7D,E) confirmed the adaptive responses of the *BdbZIP62*-expressing tobacco plants under salt and drought at the early seedling stage.

**Figure 8:**
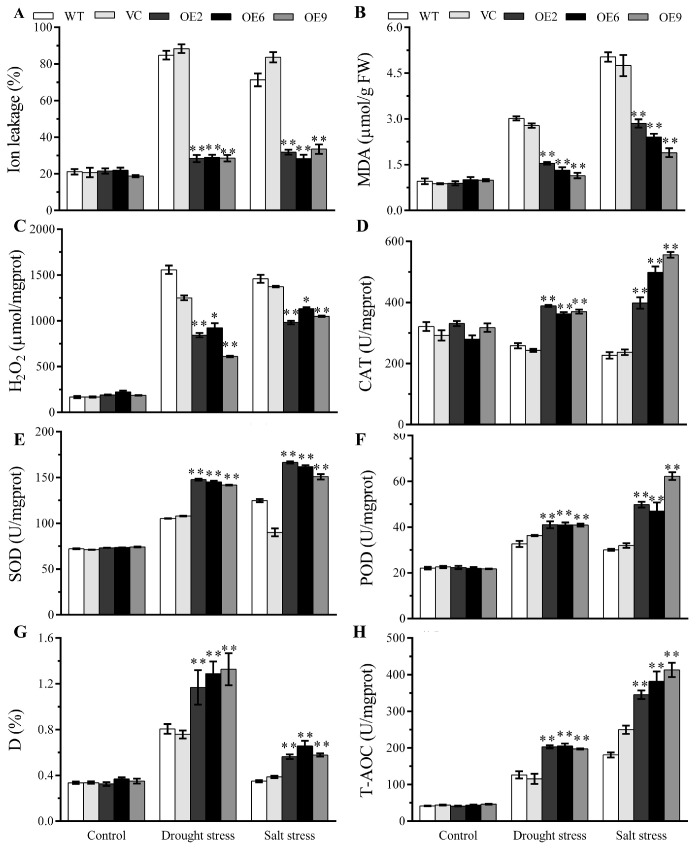
*BdbZIP62* elevated the activities of antioxidase system and alleviated the oxidative damage under drought and salt. The ion leakage (**A**), MDA content (**B**), H_2_O_2_ content (**C**), and enzyme activities of the ROS scavenging system—including CAT (**D**), SOD (**E**), and POD (**F**)—D (**G**), and T-AOC (**H**) were measured in the control and *BdbZIP62* transgenic plants under normal and stress treatments. Asterisks indicate statistically significant differences (* *p* < 0.05, ** *p* < 0.01; Student’s *t*-test).

Hence, the physiological indices related to plant tolerance were tested. Low IL, H_2_O_2_ and MDA contents, an enhanced accumulation of sugar, and increased antioxidase activities were observed in the *BdbZIP62*-expressing tobaccos compared to in the WT and VC plants, which allowed them to cope with drought and salt stresses (Figure 8 and Figure S8). However, there was no apparent difference under normal conditions. The results demonstrate that, similar to BdGF14a, the overexpression of *BdbZIP62* alleviated membrane damage due to the enhanced ROS scavenging abilities conferred by elevated T-AOC, D, POD, SOD, and CAT activities in tobacco plants under salt and drought stresses.

### 2.8. Bdbzip62 Decreases Stomatal Aperture under Abiotic Stresses and Increases ABA Sensitivity in Transgenic Tobaccos

**Figure 9:**
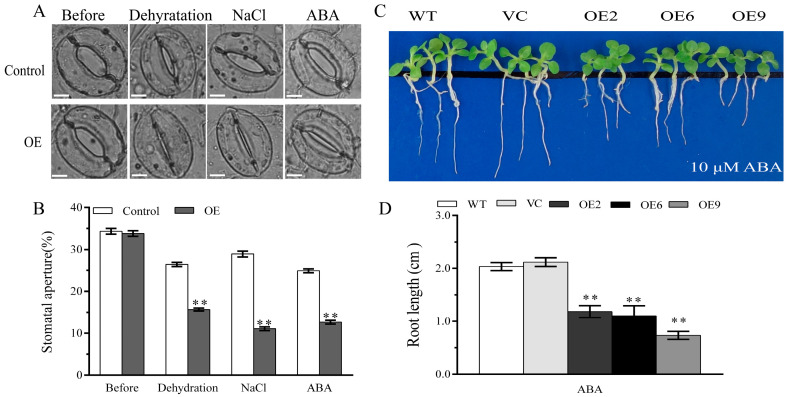
*BdbZIP62* regulates stomata movement under salt and drought treatments and ABA responses. (**A**) The stomata movements of 4-week-old *BdbZIP62* transgenic lines were detected under dehydration, 200 mM NaCl, and 50 μM ABA treatments. Scale bar = 5 μM. (**B**) Stomatal apertures were calculated under these treatments. (**C**) A photograph of root length assay in *BdbZIP62*-overexpressing tobaccos on 1/2 MS medium supplemented with 10 μM ABA for 2 weeks. (**D**) The statistical results of the root length assay. Asterisks indicate statistically significant differences (** *p* < 0.01; Student’s *t*-test).

A stomatal aperture analysis was also performed. Similar to the BdGF14a transgenics, after salinity and drought stresses, the stomata of the three *BdbZIP62* transgenic lines were almost closed with lower stomata apertures, while those of the controls were still open (Figure 9A,B). These results indicate that *BdbZIP62* also increased the pace of stomatal closure to reduce water loss, thereby participating in the response to abiotic stresses.

*BdbZIP62* is an ABRE/ABF TF family member, and it is involved in the ABRE-dependent ABA-based signaling pathway in coping with abiotic stress [32,33]. ABA is a cardinal phytohormone that functions in the response of plants to abiotic stress by regulating stomatal closure, growth, etc. [2,35,37]. As expected, the stomata of the *BdbZIP62*-expressing leaves, immersed in 50 μM ABA for 2 h, were almost closed, whereas those of most control plants remained open, with wider stomatal apertures than the transgenic plants (Figure 9A,B). In addition, the root lengths under exogenous ABA were measured to explore the ABA sensitivity of the *BdbZIP62*-expressing tobacco plants. The root lengths of the transgenic tobaccos were shorter than those of the control plants (Figure 9C,D), similar to those of the *BdGF14a* overexpression lines. These results suggest that BdbZIP62 participated in ABA-regulated stomatal closure and elevated the ABA sensitivity in tobaccos, in line with that in BdGF14a.

### 2.9. BdGF14a Enhanced the Transcriptional Regulation Activity of NtABF2 and BdbZIP62

**Figure 10:**
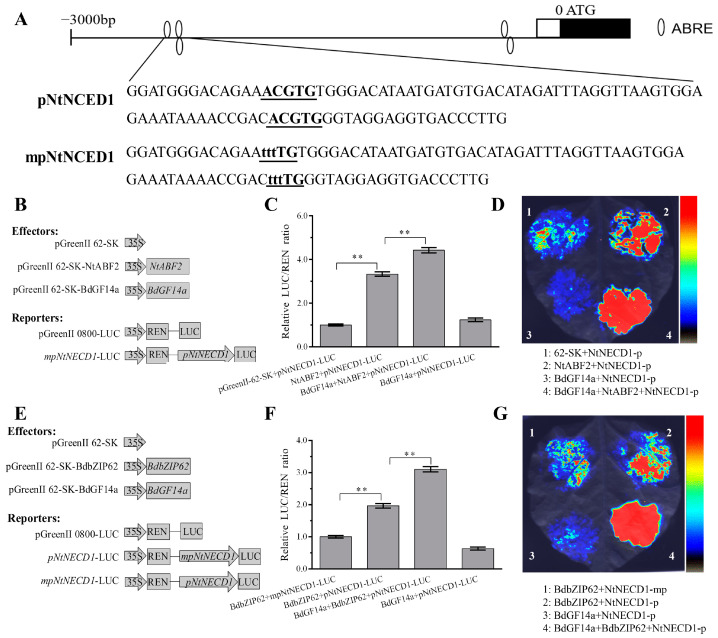
BdGF14a enhanced the transcriptional regulation activity of NtABF2 and BdbZIP62. (**A**) ABRE motif analysis of the *NtNCED1* promoter and the sequences used for the LUC assay. The fragment sequences of *NtNCED1* used for LUC assay with or without mutation are indicated in bold and underlined. (**B**) The drafts of vector constructs used for the LUC assay. The ORFs of *NtABF2* and *BdGF14a* were recombined into pGreenII 62-SK, and the sequence of NtNECD1 promoter fragment was cloned into pGreenII 0800-LUC. (**C**) The data are indicated as LUC/REN ratios. (**D**) LUC imaging assay confirmed that BdGF14a improved ability of NtABF2 to activate the transcription of *NtNCED1.* (**E**) The drafts of vector constructs used for LUC assay. The ORFs of *BdbZIP62* and *BdGF14a* were recombined into pGreenII 62-SK, and the sequence fragments of the *NtNECD1* promoter and its mutant were cloned into pGreenII 0800-LUC. (**F**) The data are indicated as LUC/REN ratios. (**G**) LUC imaging assay confirmed that BdGF14a improved the ability of NtABF2 to activate the transcription of *NtNCED1.* Asterisks indicate statistically significant differences (** *p* < 0.01; Student’s *t*-test).

NtABF2 interacted with BdGF14a by binding to the ABRE *cis*-element (Figure 6H,J), and the expression of *NtNCED1* was the most highly upregulated among the tested ABA-related genes in the BdGF14a-expressing tobaccos under abiotic stress (Figure 6I). The ABRE *cis*-element was identified in the 5′-UTR upstream region of the *NtNCED1* gene (Figure 10A). A dual-luciferase reporter (LUC) assay was performed to validate the transcriptional regulation activation of NtABF2 and the affection of BdGF4a to the binding. The 94 bp promoter fragment of *NtNCED1* used as the target is shown in Figure 10A. The drafts of the vector constructs used for the LUC assay are shown in Figure 10B. The LUC/REN ratio was ascertained to evaluate the transcriptional activity, and the co-transformed pGreenII 62-SK empty vector and *pNtNECD1*-LUC, taken as a control, were set to 1. The results show that NtABF2 could activate *pNtNECD1*-LUC (Figure 10C,D). Moreover, the coexpression of pGreenII 62-SK-BdGF14a and pGreenII 62-SK-NtABF2 with *pNtNECD1*-LUC conspicuously improved the LUC/REN ratio compared with the expression of pGreenII 62-SK-NtABF2 alone (Figure 10C,D). These observations revealed that *BdGF14a* could increase the transcription regulation activity of NtABF2, indicating that NtABF2 could indeed activate the expression of *NtNECD1* by binding with the ABRE *cis*-element in its promoter, and BdGF14a could promote the activity of NtABF2 by interacting with it. These results demonstrate that *BdGF14a* improved salt and drought resistance by binding to NtABF2 and enhancing its transcriptional regulation activity to upregulate the expression of genes with the ABRE *cis*-element related to the ABA signaling pathway.

BdbZIP62 is the ortholog of NtABF2 in *B. distachyon.* Hence, it was ascertained whether BdbZIP62 could regulate transcription activity and whether BdGF4a could affect this ability. Therefore, a Y1H assay was carried out to detect the regulatory action of BdbZIP62 to the ABRE *cis*-element. The yeast cells co-transformed with the ABRE *cis*-element and BdbZIP62 had a better growth status on SD-LWH + 100 mM 3-AT than those containing empty vectors (Figure S9). Moreover, a LUC assay further validated the interaction of BdbZIP62 and the promoter of NtNCED1 and the affection of BdGF4a to the bonding. The mutant sequence of the *NtNCED1* promoter in the ABRE cis-element used as the target is listed in Figure 10A, and the drafts of the vector constructs used for the LUC assay are shown in Figure 10E. The LUC/REN ratio of the cells co-transformed with pGreenII 62-SK-BdbZIP62 and *mpNtNECD1*-LUC was set to 1 and taken as the control. These results show that BdbZIP62 could activate *pNtNECD1*-LUC, and coexpressing pGreenII 62-SK-BdGF14a and pGreenII 62-SK-BdbZIP62 with *pNtNECD1*-LUC resulted in a higher LUC/REN ratio than expressing pGreenII 62-SK-BdbZIP62 alone (Figure 10F,G), suggesting that, similar to NtABF2, the coexpression of BdGF14a could also increase the transcription regulation activity of BdbZIP62 targeting *NtNECD1* by binding with its ABRE *cis*-element. However, a lower LUC/REN ratio was detected in *mpNtNECD1*-LUC (lacking an ABRE *cis*-element) pairs (Figure 10C,D). The above results indicate that the transcription activation activity of BdbZIP62 to *NtNECD1* was partly ABRE *cis*-element-specific, and BdGF4a could also promote this activity by interacting with BdbZIP62.

## 3. Discussion

The response of plants when facing various kinds of abiotic stress, including drought and salinity, involves complex signal transduction networks [1,2]. 14-3-3s are reported to exert critical functions in the response of plants to adverse environments [18,19,31,42]. Herein, a 14-3-3 was identified from *B. distachyon*, a monocot model plant closely related to triticeae crops. It was designated BdGF14a due to its high similarity of 99% to HvGF14a (Figure S1), which was named BdGF14f based on the homology of rice 14-3-3s used in previous studies [31,42]. An analysis of the subcellular localization suggested that BdGF14a was detectable in the whole onion cell (Figure S3), similar to OsGF14f, the most homologous gene in rice, and BdGF14d in *B. distachyon* [24,32]. *BdGF14a* was upregulated by NaCl, H_2_O_2_, and ABA (Figure S2), illustrating its potential function in response to these stresses [31,42]. Furthermore, the *BdGF14a* transgenic tobacco plants were first demonstrated to have an enhanced salinity- and drought-tolerant phenotype at the early and late seedling stages, with a better growth status, a higher survival rate, and longer roots (Figure 1). Somewhat similar, OsGF14f and HvGF14a, two genes with the closest evolutionary relationship with *BdGF14a* in barley and rice, are involved in drought resistance [20,24], reflecting that they might be a suitable and efficient path for the rapid mining and parsing of genes from *B. distachyon*. Thus, it is hypothesized that OsGF14f and HvGF14a might play a potential role in the salt response, and our study offers a novel target for the breeding of salt- and drought-tolerant plants.

Abiotic stress leads to the overaccumulation of ROS, which can damage plant cells [43,44]. GPX, APX, SOD, and CAT detoxify ROS [43]. In this study, the activities of CAT, SOD, POD, T-AOC, and D were increased in the *BdGF14a*-expressing tobaccos (Figure 3A). Similarly, the transcription of ROS-related genes, including *NtCAT*, *NtSOD*, *NtPOX2*, *NtGST*, *NtAPX*, *NtRBOHF*, and *NtRBOHD*, was higher in the *BdGF14a*-expressing tobacco lines than in the control plants (Figure 4). Thus, a lower H_2_O_2_ content was detected (Figure 3A). These results were further confirmed via NBT and DAB staining. A darker color was observed in the leaves of the WT and VC plants than in those of the *BdGF14a*-expressing tobaccos under stress treatments, indicating a lower production of ROS (O_2_^−^ and H_2_O_2_) in the transgenics in vivo (Figure 3B). These results demonstrate that *BdGF14a* enhanced the antioxidant capacity by improving the ROS scavenging system under drought and salt treatments. In addition, much greener leaves and a higher chlorophyll content were observed in the *BdGF14a*-expressing tobaccos in an MV solution (Figure S4), revealing that *BdGF14a* was involved in the response to oxidative-state-related stress.

The stability of the membrane was reduced and the ion balance was disturbed under stress treatment. Thus, lower IL (an index of membrane injury) and MDA (used for evaluating defects in ROS-mediated lipid peroxidation in plant cells) were detected in the *BdGF14a* transgenics under salt and drought stress than in the control tobaccos (Figure 2A,B). Additionally, higher osmoprotectant (sugar and/or proline) contents were also detected in the *BdGF14a* transgenic tobaccos (Figure 2C,D) to ease the cell damage caused by salinity- and drought-induced stresses. These results agree with the upregulation of the expression of *NtSUS1*, *NtSPSA*, and *NtP5CS1*, which participated in the biosynthesis of sucrose and proline under salinity and/or drought treatments (Figure S5) [45]. Moreover, SOS1, as a membrane-bound Na^+^/H^+^ antiporter, improved salt tolerance [12,46], while two antiporters, NHX2/4, balanced the ion levels in plant cells [47,48], and CAX regulated Ca^2+^ homeostasis [49,50]. A 14-3-3 protein from *B*. *distachyon*, BdGF14d, regulated ion transporters at the transcriptional level [32]. Therefore, the upregulation of these genes (*NtSOS1*, *NtNHX2*/*4*, *NtCAX3*/*2*, and *NtNKT1*) in the *BdGF14a* transgenic lines compared to WT under salinity and drought (Figure S6) suggests that BdGF14a could regulate ion transport to avoid toxicity and maintain the stability of the membrane in coping with these conditions. The above results demonstrate that BdGF14a regulated the accumulation of osmotic compounds and the transport of ions to enhance the stability of the membrane under abiotic stresses.

ABA has been identified to exert a cardinal function in coping with abiotic stresses [2,37,38], participating in stomatal closure, growth regulation, and the inhibition of seed germination [33,35,51]. *BdGF14a* (Bradi1g11290) could be upregulated by exogenous ABA (Figure S2F) [31,32,42]. ABRE/ABF/ABI5, the group A subfamily of bZIP TFs, are prominent regulators of the ABA signaling pathway and abiotic stress responses [33]. 14-3-3s interacted with ABRE/ABF TFs and responded to adverse environments through the ABA-related signaling pathway [21,32,36]. Similar to the reports of the interactions of MdGRF11 and OsGF14f with ABRE/ABFs in coping with abiotic stresses [23,52,53], BdGF14a interacted with NtABF2 (Figure 6H). The root lengths were shorter in the *BdGF14a*-expressing tobaccos than in the control tobaccos when treated with exogenous ABA (Figure 5C,D). However, under both drought and salt treatments, these differences disappeared when Tu was added (Figure 5C,D). Furthermore, as with HvGF14A [20], the stomata of the *BdGF14a* expression tobaccos closed more quickly during drought, salt, and exogenous ABA treatments (Figure 5A,B). These findings imply that BdGF14a enhanced the sensitivity to ABA and might participate in ABA-associated signaling. In *Arabidopsis*, ABI5 functioned in ABA signaling and ROS homeostasis during seed germination [54]. This investigation showed that the upregulation of ROS-scavenging-related genes, such as *NtCAT*, *NtSOD*, and *NtPOX2*, was suppressed when Tu was added (Figure 4) under drought and salt treatments. These results also follow reports suggesting that ABA could upregulate the transcription of antioxidant-system-related genes and enhance the activities of ROS scavenging enzymes for alleviating oxidative damage [29,55,56,57,58]. As in the case of the genes involved in ROS scavenging, the expression of most other stress-related genes was also inhibited when Tu was added (Figures S5 and S6). Hence, these results demonstrate that BdGF14a enhanced the resistance to abiotic stress via the participation of ABA-based signaling.

Moreover, the overexpression of *MdGRF11* improved drought tolerance by altering the transcription of marker genes related to ABA signaling [53]. OsGF14f positively mediated the ABA-based responses induced by drought via interaction with OsbZIP23, a notable ABRE/ABF TF, to activate the transcriptional regulation of downstream target genes by binding to the ABRE elements of their promoters [23,24,40,41]. Hence, the transcription of the ABA-related genes *NtDREB3*, *NtERD10C/D*, *NtTobLTP1*, *NtLTP1*, and *NtLEA5*, which functioned in coping with abiotic stress, was selected to analyze the molecular mechanisms more thoroughly [59,60,61], which were remarkably increased in the OE tobaccos under salt and drought (Figure 6A–F). In addition to *NtNCED1*, a crucial ABA biosynthesis-related gene, *NtABF2*, encoding a critical transcriptional regulator of ABA-based signaling, was also highly expressed (Figure 6G,I) [62,63]. TabZIP60 directly binds to the promoter of *TaNCED2* at the ABRE core sequences to respond positively to salt-induced stress with a higher ABA content [40]. OsbZIP23 promoted the expression of *OsNCED4*, a vital gene related to ABA biosynthesis, by combining with the ABRE element of its promoter to mediate the feedback regulation of the ABA response, and the “OsbZIP23-OsGF14f” interaction enhanced this transcriptional activity while coping with osmotic-state-related stress [24]. In barley, HvABI5 regulated the response to drought and the transcription of *HvNCED1*, a putative direct target gene, by bonding to the ABRE element of the promoter induced in *hvabi5* [64]. Since *NtNCED1* was significantly upregulated in the *BdGF14a*-expressing tobaccos after different treatments and its promoter possessed the ABRE element (Figure 10A), it was deduced that it, too, was a putative direct target of NtABF2. Hence, Y1H and LUC assays were carried out; as expected, NtABF2 interacted with the ABRE cis-element (Figure 6J), and BdGF14a enhanced the ability of NtABF2 to upregulate the expression of *NtNCED1* (Figure 10B–D), which might act as a positive feedback regulator of ABA accumulation and improve the ABA-dependent resistance to salinity and drought conferred by BdGF14a.

As reported in prior research by our group, BdbZIP62, an orthologous protein of NtABF2 and an ABI5-like protein belonging to the BdABRE/ABF family, interacted with all Bd14-3-3s, including BdGF14a [32]. BdbZIP62 was considered the first to provide further insight into the mechanism by which BdGF14a responded to abiotic stresses in *B. distachyon* by confirming its function in regulating the resistance to abiotic stresses. The mutants of *AtABF3* and *AtABF4* displayed sensitivity to ABA, salt, and drought; the heterologous expression of *AtABF3* conferred improved tolerance to salinity and dehydration in alfalfa [65,66]. *OsABF2*, *OsbZIP23*, and *ABP9*, encoding a maize ABRE/ABF TF, regulated the response to drought and salt through an ABA-dependent pathway in mutation or overexpression experiments [52,67,68]. *ZmABP9* transgenic cotton lines were more tolerant to stress by altering the width of the stomatal aperture, the expression of stress-related genes, and physiological indices (including lowered ROS production, higher activities of detoxifying enzymes, and increased production of proline and soluble sugars) while coping with salt and osmotic stresses [68]. In the present research, the transcription of *BdbZIP62* was induced by treatments with PEG, NaCl, H_2_O_2_, and ABA (Figure S7), indicating that it was responsive to drought and salt. Additionally, *BdbZIP62* conferred resistance to drought and salinity in tobaccos at both the early and late seedling stages, with physiological indices similar to those of *BdGF14a.* These were characterized by a better growth status, a higher survival rate, less water loss, and longer root lengths (Figure 7); an enhanced speed of stomatal closure (Figure 10A,B); and altered physiological indices, such as a higher soluble sugar content (Figure S8), reduced IL, MDA, and H_2_O_2_ production, and enhanced ROS scavenging abilities (Figure 8). Furthermore, *BdbZIP62* also decreased the width of the stomatal apertures and the root length of the transgenic plants exposed to ABA (Figure 9). Similarly to the interaction of BdGF14a–NtABF2, BdGF4a also promoted the transcriptional activation activity of BdbZIP62 by bonding to the ABRE *cis*-element in the promoter of *NtNECD1* (Figure 10 and Figure S9). OsbZIP23 and OsGF14f conferred better drought resistance in rice via the feedback regulation of ABA signaling and biosynthesis [23,40,41]. OsGF14f interacted with OsbZIP23 and was a direct regulatory target of OsbZIP23; OsGF14f positively regulated the response of OsbZIP23 to osmotic-state-related stress; and OsGF14f-induced drought resistance partially depended on the feedback regulation of OsbZIP23 in combination with the ABRE motifs of the *OsNCED4* promoter [24]. Therefore, it can be speculated that the resistance to drought and salinity conferred by BdGF14a and BdbZIP62 may depend on their interaction, which regulates the ABA signaling pathway and the functioning of BdGF14a via feedback. However, a more exact understanding of the function and the underlying molecular mechanism of the “BdGF14a-BdbZIP62” interaction needs experimental verification. Our work provides new insights into the role of the “BdGF14a-BdbZIP62” regulatory module in *B. distachyon* under drought- and salt-induced stresses.

## 4. Materials and Methods

### 4.1. Plant Materials and Stress Treatments

Sterilized *B. distachyon* seeds were spread on moist filter paper in Petri dishes for germination and culture under a 16 h light/8 h dark cycle at 150 µmol photons m^−2^ s^−1^ and 25 °C for two weeks. The seedlings with a similar growth status were selected for treatment with 10 mM H_2_O_2_, 100 μM ABA, 200 mM NaCl, and 20% PEG6000. The leaves of the treated seedlings were collected at 0, 1, 3, 6, 12, and 24 h of treatment. Additionally, tissues, including roots, stems, leaves, and spikelets, of *B. distachyon* plants cultivated in pots were also obtained. The samples were stored at −80 °C.

*Nicotiana tabacum* L. (Samsun) was used for genetic transformation and stress tolerance analyses. The T_3_ seeds of the *BdGF14a* and *BdbZIP62* transgenic tobacco plants, VC plants, and WT plants were sterilized following the instructions mentioned in a previous report [29]. The sterilized seeds were germinated on half MS medium without vitamins in Petri dishes in a growth chamber under a 16 h/8 h dark cycle at 120 µmol photons m^−2^ s^−1^ and 22 °C. The two-week-old tobacco plants were then potted in vermiculite and grown in phytotron.

### 4.2. Cloning of BdGF14a and Bioinformatic Analyses

Using gene-specific primers, *BdGF14a* was cloned from a mixture of RNA extracted from the *B. distachyon* seedlings after treatment with ABA, H_2_O_2_, PEG6000, and NaCl (Table S1). For the phylogenetic analysis of BdGF14a, the homologs, including eight 14-3-3s from *Triticum aestivum*, eight from *Oryza sativa*, HvGF14a, and AtGF14a, were accessed from NCBI. Then, the construction of the phylogenetic tree and sequence alignment of the BdGF14a homologs were performed using MEGA5.0 and ClustalX [29].

### 4.3. Subcellular Localization Analyses

The ORF of *BdGF14a* was cloned into *pBI121*-*GFP* (VC), driven by the CaMV35s promoter. The recombinant and VC plasmids were then separately packed with gold powder and used to bombard the onion epidermal cells based on a previously reported method [29].

### 4.4. Tobacco Transformation and Stress Tolerance Analyses

The *pBI121*-*BdGF14a-GFP* and *pBI121*-*BdbZIP62-GFP* plasmids were used to transform the tobacco plants using the *Agrobacterium*-mediated transformation method [69]. On a medium containing 100 mg/L kanamycin (Kan), three stable independent transgenic lines for each gene were screened, and their transcript levels were ascertained using qRT-PCR. The T_3_ seeds of the *BdGF14a* and *BdbZIP62* transgenic tobacco plants were harvested for abiotic stress tolerance assays.

To detect the changes in root length under different stresses, the seeds of the three *BdGF14a*- or *BdbZIP62*-overexpressing lines and control plants were first germinated on a conventional medium and then transferred to half MS medium with or without 100 mM NaCl, 150 mM mannitol, or 10 μM ABA. The *BdGF14a* transgenic plants were transplanted to a medium with 100 mM NaCl + 0.1 mM Tu or 150 mM mannitol + 0.1 mM Tu. After vertical cultivation for 2 weeks, the root lengths of these seedlings were measured. For drought treatment, 5-week-old tobacco plants were exposed to dehydration for 6 weeks and re-watered for 3 weeks. For salt treatment, the seedlings with a similar growth status were irrigated with 500 mM salt solution for 4 weeks. The survival rates of these lines were measured following these treatments.

The seeds of the *BdGF14a*-expressing and WT plants were germinated on a medium to examine the oxidative tolerance conferred by *BdGF14a*. After 3 weeks of growth, the leaves of all tobacco lines were collected and then stained in distilled water with or without 10 µM MV for 96 h. Images were then captured, and the chlorophyll content was measured.

### 4.5. Measurement of the Physiological Parameters Indicative of Drought and Salt Responses

To ascertain the physiological indices under stress and typical environments, the leaves of the *BdGF14a* or *BdbZIP62* transgenic lines and control plants were collected and ground in PBS on ice. The supernatant was obtained after centrifugation at 4500 RCF for 20 min. The sugar, proline, H_2_O_2_, and MDA contents and the activities of the antioxidant enzymes CAT (EC 1.11.1.6), SOD (EC 1.15.1.1), and POD (,EC 1.11.1.7), along with T-AOC and D, were measured using specific kits (Comin, Suzhou, China). IL was also measured using a previously reported method [55]. The following formula was used to calculate IL: IL (%) = C1/C2 × 100.

### 4.6. DAB and NBT Staining Assays

DAB and NBT staining experiments of the *BdGF14a* transgenic plants were conducted by referring to a previously reported method [29]. After 2 days of treatments with 300 mM mannitol and 200 mM NaCl, the whole seedlings of the treated and control groups were stained overnight in 0.1 mg/mL NBT and 1 mg/mL DAB dyeing solutions at 37 °C, respectively. The control and three *BdGF14a* transgenic seedlings were moved to a solution of glycerol, glacial acetic acid, and absolute ethyl alcohol at a ratio of 1:1:3 (*v*/*v*) and boiled for 20 min or until the extraction of all the chlorophyll of the leaves. The results were recorded, and images were subsequently captured. In addition, after the exposure of the four-week-old tobaccos growing in sand to 500 mM NaCl for 3 weeks and dehydration for 1 week, the leaves were obtained and immersed in DAB and NBT staining solutions by following the method mentioned above.

### 4.7. Stomatal Closure Assay

A stomatal closure assay was performed per a previous report [29]. The leaves of the 4-week-old tobacco (WT, VC, and OEs) plants were immersed in a solution containing 50 μM CaCl_2_, 10 mM MES-KOH, and 10 mM KCl (pH ± 6.15) and incubated under a high-intensity light of 200 μmol m^−2^ s^−1^ for 4 h to open the stomata. Then, the leaves were removed either for dehydration at room temperature for 40 min or immersion in the solution with 50 μM ABA for 1 h or 200 mM NaCl for 30 min. After treatment, the stomata were observed under an IX71 microscope (Olympus, Tokyo, Japan).

### 4.8. qRT-PCR Assays

The 2-week-old seedlings were grown on a medium containing 300 mM mannitol or 200 mM NaCl with or without 0.1 mM Tu for five days and were harvested to determine the transcript levels of stress-related genes using qRT-PCR. The expression of stress-responsive genes was determined, including that of the ABA-related genes *NtABF2*, *NtNCED1*, *NtLEA5*, *NtDREB3*, *NtLTP1*, *NtTobLTP1*, *NtERD10C*, and *NtERD10D*; that of the ROS-scavenging-related genes *NtCAT*, *NtSOD*, *NtPOX2*, *NtGST*, *NtAPX*, *NtRBOHD*, and *NtRBOHF*; that of the ion-channel-related genes *NtSOS1*, *NtCAX3*, *NtCAX2*, *NtNKT1*, and *NtNHX4/2*; and that of other stress-related genes, namely, *NtSPSA*, *NtSUS1*, *NtADC1*, *NtSAMDC*, and *NtP5CS1*. The RNA of the samples was extracted using an RNA extraction kit (Zoman, Beijing, China), and the cDNA was obtained using a FastQuant RT kit (Tiangen, Beijing, China). Detailed information of the gene-specific primer pairs is given in Table S2 [29,32]. The 2^−ΔΔCT^ method was used to analyze the data and compute the relative expression levels [70].

### 4.9. Y2H Assay

A Y2H assay detects the interaction between BdGF14a and NtABF2 when applying the manufacturer’s protocol (Clontech, USA). The full-length *BdGF14a* coding sequence was cloned into the BD vector, and NtABF2 was cloned into the AD vector. Next, these constructs were used to co-transform the yeast strain AH109; plated on a SD-2 selection medium for 2–3 d; spotted on nutritional selective solid media SD-2, -3, and -4; and incubated at 30 °C for 3–5 d. The transformants of *pGADT7-T* or *pGBKT7-p53* and *pGBKT7-LaminC* were used as the positive and negative controls, respectively. Detailed information on the gene-specific primers used is presented in Table S3.

### 4.10. Y1H Assay

Three tandem repeat ABRE motif sequences were cloned into pHIS2, and the ORF of BdbZIP62 was cloned into pGADT7. Then, the recombinant vectors *pHIS2*-*ABRE* and pGADT7-*NtABF2*/*BdbZIP62* were used to transform the yeast strain Y187, and cells with *pGADT7*-*NtABF2* were plated on an SD-LW selection medium at 30 °C for 2–3 d. They were then spotted on the nutritional selective solid medium SD-LW or SD-LWH with or without 100 mM 3-AT and incubated for another 3–5 d. The plants co-transformed with the pHIS2-ABRE + pGADT7 empty vector and pGAD53m + pHIS2-P53 were used as the negative and positive controls, respectively. The interaction was estimated by the growth ability of the yeast cells on selective plates SD-LWH + 100 mM 3-AT. The relevant primers are listed in Table S3.

### 4.11. LUC Assay

A LUC assay was conducted following a previously reported protocol [71]. The ORFs of NtABF2, BdbZIP62, and BdGF14a were inserted into pGreenII 62-SK, and the f NtNECD1 promoter fragment sequence and its mutant were cloned into pGreenII 0800-LUC via homologous recombination and synthesis at Sangon (Sangon, Shanghai, China), respectively. The plasmids were then transformed into the *Agrobacterium tumefaciens* strain GV3101-pSoup and co-infiltrated into the tobacco leaves using a previously described transient expression method [16]. After infiltration in a growth chamber for 72 h, the luminescence activity was detected using a Glomax 2020 imaging system (Promega, Madison, WI, USA). The primers and sequences used are listed in Table S4.

### 4.12. Statistical Analyses

GraphPad Prism 6.01 software (GraphPad Inc., San Diego, CA, USA) was used for statistical analyses with Student’s *t*-test. Standard error bars were calculated based on at least three independent replicate experiments.

## 5. Conclusions

In conclusion, this is the first study to verify that BdGF14a and BdbZIP62 confer resistance to drought and salt in tobacco, characterized by a higher survival rate, a longer root length, enhanced cell membrane stability, elevated activities of detoxifying enzymes, rapid stomatal closure, and/or the expression of stress-related genes, through the regulation of ABA-based signaling. BdGF4a also promoted the transcriptional activation activity of BdbZIP62 by binding to the ABRE *cis*-element of the *NtNECD1* promoter, which was similar to the function of BdGF4a to NtABF2. The interaction of BdGF14a with BdbZIP62, the similarity of the associated phenotypes, and the indicators of physiology and biochemistry pave a novel way for understanding the GF14s-bZIPs-NCED-based regulatory module against abiotic stresses. These findings can provide direction for subsequent research that can be conducted on this protein–protein interaction to deeply analyze the role and underlying mechanism of BdGF14a and BdbZIP62 in functioning under drought and salt environments.

## Data Availability

The data presented in this study are available upon request from the corresponding author.

## Supplementary Materials

The following supporting information can be downloaded at https://www.mdpi.com/article/10.3390/plants13020245/s1, Figure S1: Phylogenetic relationship and multiple alignments of BdGF14a with other GF14 members; Figure S2: Expression patterns of BdGF14a in different organs and stress treatments in *B. distachyon* analyzed using qRT-PCR analysis; Figure S3: Subcellular localization of GFP and BdGF14a::GFP fusion protein in onion epidermal cells; Figure S4: Oxidative tolerance analysis of three *BdGF14a*-overexpressing and WT tobaccos under MV treatment; Figure S5: Expression patterns of osmolytes (polyamine, soluble sugar, and proline) biosynthesis-related genes in BdGF14a transgenic tobaccos under stress-inducing conditions; Figure S6: Expression patterns of ion channel-related genes in BdGF14a transgenic tobaccos under normal and stress-inducing environments; Figure S7: Expression patterns of *BdbZIP62* in different organs and stress treatments determined using qRT-PCR analysis in *B. distachyon*; Figure S8: The sugar contents in BdbZIP62 transgenic tobaccos under salinity and drought; Figure S9: Y1H assay of BdbZIP62 interacted with ABRE *cis*-element. Table S1: Primers for qRT-PCR and construction pBI121 vector of *BdGF14a* and *BdbZIP62*; Table S2: Primers for ascertaining the expressions of stress-related marker genes; Table S3: Primers for Y1H andY2H assays; Table S4: Primers and sequences for LUC assay.
