# Supplementary material for: The 14-3-3 Protein BdGF14a Increases the Transcriptional Regulation Activity of BdbZIP62 to Confer Drought and Salt Resistance in Tobacco
Source: Plants (Basel). 2024 Jan 15;13(2):245. doi: 10.3390/plants13020245 (PMC10819667; doi:10.3390/plants13020245)
Supplement: Supplementary file 1 [file plants-13-00245-s001.zip › Supplementary Materials.pdf]

Table S1. Primers for qRT-PCR and constructions pBI121 vector of *BdGF14a* and *BdbZIP62*.

Gene Name	Forward/ Reverse primers(5′-3′)
BdGF14a-qPCR	CATTTACGCAGCTACCTGT
	AATGGACTAATAACGCAGCTC
BdZIP62-qPCR	GAGAGGCAAAAGAATGAG
	AGACATCTTCAGTCACGG
BdSamDC	TGCTAATCTGCTCCAATGGC
	GACGCAGCTGACCACCTAGA
pBI121-*BdGF14a*	GCTCTAGAATGTCTACTGCTGAGGCAACC
	CGGGATCCGTGACCCTCTCCTTCAGGC
pBI121-*BdZIP62*	GCTCTAGAATGGATTTTCCGGGAGGGAG
	CGGGATCCCGGCCCTGTCAGTGTCC

Table S2. Primers for ascertaining the expressions of stress-related marker genes.

Gene Name	Forward/ Reverse primers(5′-3′)
NtGAPDH	TGCCTTGAGCAAGAACTTTGtG
	GGCAGATCAAATCAATCACACG
NtERD10D	GAGGACACGGCTGTACCAGT
	GCGCCACTTCCTCTGTCTT
NtERD10C	AACGTGGAGGCTACAGATCG
	GTTCCTCTTGGGCATGAGTT
NtLEA5	TTGAATCTGGGGTTTTGGTT
	GGAAGCATTGACGAGCTAGG
NtNCED1	AAGAATGGCTCCGCAAGTTA
	GCCTAGCAATTCCAGAGTGG
NtABF2	GCAGCCATCTATCTATTC
	GCAACTCATCCATATTCA
TobLTP1	GGTTTTGTGCATGGTGGTGG
	CTTAGAGCAGTCTGTGGAGG
NtLTP1	GCAGAAGCCATAACCTGTGG
	CAGTGGAAGGGCTGATCTTG
NtDREB3	GCCGGAATACACAGGAGAAG
	CCAATTTGGGAACACTGAGG
NtPOX2	CTTGGAACACGACGTTCCTT
	TCGCTATCGCCATTCTTTCT
NtSOD	CTCCTACCGTCGCCAAAT
	GCCCAACCAAGAGAACCC
NtCAT1	AGGTACCGCTCATTCACACC

	AAGCAAGCTTTTGACCCAGA
NtAPX	CAAATGTAAGAGGAAACTCAGAGGA
	CAGCCTTGAGCCTCATGGTACCG
NtGST	CCCCTAGTTTGCTCCCTTCT
	TTCTTAGCTGCCTCCTGCTC
NtCAX3	CGGTTTGGCAATAATTGTCACAG
	CAACGATCATGCTTCAATCATCC
NtCAX2	GGTCATCAACTCAGATAT
	TAGCATAGAATAAGCATCA
NKT1	CCTAATGAATCTGATAGC
	CGAGAAGTAGAAGAATAC
NtSOS1	CAAATGTTATCCCCCGAAAGC
	CGGAGAACCTGAGGAAATGTGA
NtNHX2	ACTCATCCCCATTGGTCCG
	AAGGAGTTCCACAAAAGCACGA
NtNHX4	CAAGAACTTCCGCACCCAC
	GCAGTATCAAACGCAGAGGACC
NtRbohD	AATAAGGAGTCTAAGGAAT
	GGAATCAGTTGTAATGTT
NtRbohF	AATAATGGTAATCCTTATGG
	GTAGACAATGACAAGAAG
NtP5CS1	ATCTTCTAGTTCTGTTGA
	CTCTCCTTAATGTATGTG
NtADC1	CTTGCTGATTACCGCAATTTATC
	TAGGATCAGCAGCCCCCATAGCC
NtSAMDC	CATTCACATTACCCCGGAAG
	AGCAACATCAGCATGCAAAG
NtSUS1	GTGGGGAAACACCGCTGAA
	CAACAAGGATGCGAGGGATGA
NtSPSA	GAATTCAGGCGCTTCGTTGTCA
	ACCCCTAGTTTCTCCAGTGA

Table S3. Primers for Y1H and Y2H assays.

Gene Name	Forward/ Reverse primers(5′-3′)
pGBKT7-BdGF14a	GGAATTCCATATGATGTCTACTGCTGAGGCAACC
	CGGGATCCCTTAGTGACCCTCTCCTTCAGGC
pGADT7-NtABF2	CCATGGAGGCCAGTGAATTCATGGGGAGTAATTTTAATT
	CAGCTCGAGCTCGATGGATCCTTACCATGGACCAGTCTG
pHIS2-ABRE	AATTCACGTGACGTGACGTGGAGCT
	CCACGTCACGTCACGTG

Table S4. Primers and sequences for LUC assay.

Gene Name	Forward/ Reverse primers(5′-3′)
pGreenII 62-SK-NtABF2	TAGAACTAGTGGATCCATGGGGAGTAATTTTAATTTC
	CGGGCCCCCCCTCGAGTTACCATGGACCAGTCTGTCTG
pGreenII 62-SK-BdbZIP62	TAGAACTAGTGGATCCATGGATTTTCCGGGAGGGAG
	CGGGCCCCCCCTCGAGTCACGGCCCTGTCAGTGTCC
pGreenII 62-SK-BdGF14a	TAGAACTAGTGGATCCATGTCTACTGCTGAGGCAA
	CGGGCCCCCCCTCGAGGTGACCCTCTCCTTCAGGC
pGreenII 0800-LUC-NtNECD1-p	CGGGCCCCCCCTCGAGGGATGGGACAGAAA
	TAGAACTAGTGGATCCCAAGGGTCACCTC
pGreenII 0800-LUC-NtNECD1-mp	CGGGCCCCCCCTCGAGGGATGGGACAGAATTT
	TAGAACTAGTGGATCC CAAGGGTCACCTC
NtNECD1-p	GGATGGGACAGAAACGTGTGGGACATAATGATGTGACATAGATTTAGG
	TTAAGTGGAGAAATAAAACCGACACGTGGGTAGGAGGTGACCCTTG
NtNECD1-mp	GGATGGGACAGAAtttTGTGGGACATAATGATGTGACATAGATTTAGGTT
	AAGTGGAGAAATAAAACCGACtttTGGGTAGGAGGTGACCCTTG

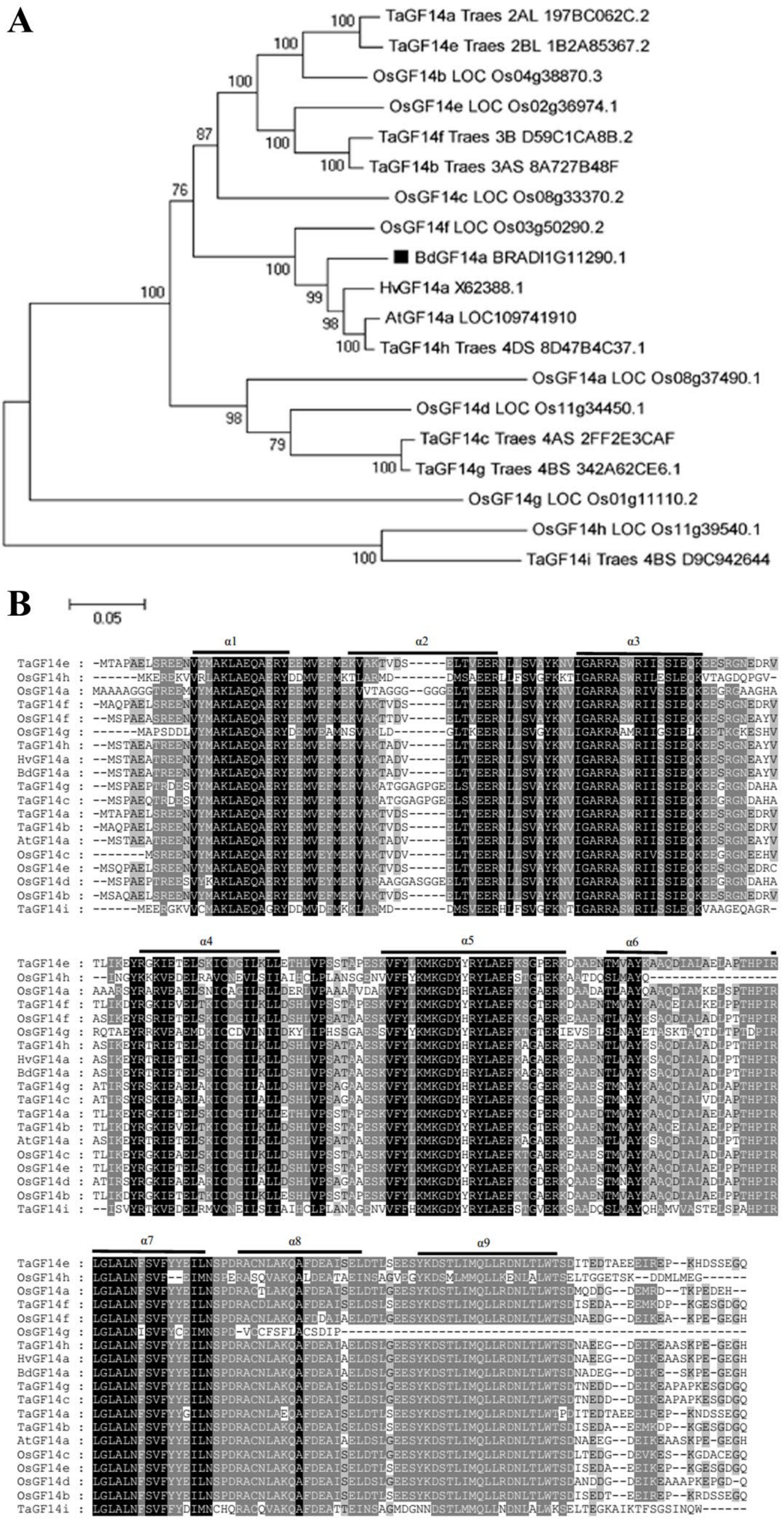
A
TaGF14a Traes 2AL 197BC062C.2
TaGF14e Traes 2BL 1B2A85367.2
OsGF14b LOC Os04g38870.3
OsGF14e LOC Os02g36974.1
TaGF14f Traes 3B D59C1CA8B.2
TaGF14b Traes 3AS 8A727B48F
OsGF14c LOC Os08g33370.2
OsGF14f LOC Os03g50290.2
BdGF14a BRADI1G11290.1
HvGF14a X62388.1
AtGF14a LOC109741910
TaGF14h Traes 4DS 8D47B4C37.1
OsGF14a LOC Os08g37490.1
OsGF14d LOC Os11g34450.1
TaGF14c Traes 4AS 2FF2E3CAF
TaGF14g Traes 4BS 342A62CE6.1
OsGF14g LOC Os01g11110.2
OsGF14h LOC Os11g39540.1
TaGF14i Traes 4BS D9C942644
0.05
B
α1
α2
α3
α4
α5
α6
α7
α8
α9

Figure S1. Phylogenetic relationship and multiple alignment of BdGF14a with other GF14 members. (A) Phylogenetic relationship of BdGF14a with TaGF14a (2AL), TaGF14e (2BL), OsGF14b, OsGF14e, TaGF14f, TaGF14b, OsGF14c, OsGF14f, HvGF14a, AtGF14a, TaGF14h, OsGF14a, OsGF14d, TaGF14c, TaGF14g, OsGF14g, OsGF14h and TaGF14i was constructed by MEGA6. (B) Multiple of BdGF14a protein. Multiple alignment of amino acid sequences BdGF14a and other related GF14 proteins from selected plant species. Alignments were performed by using Clustal W software.

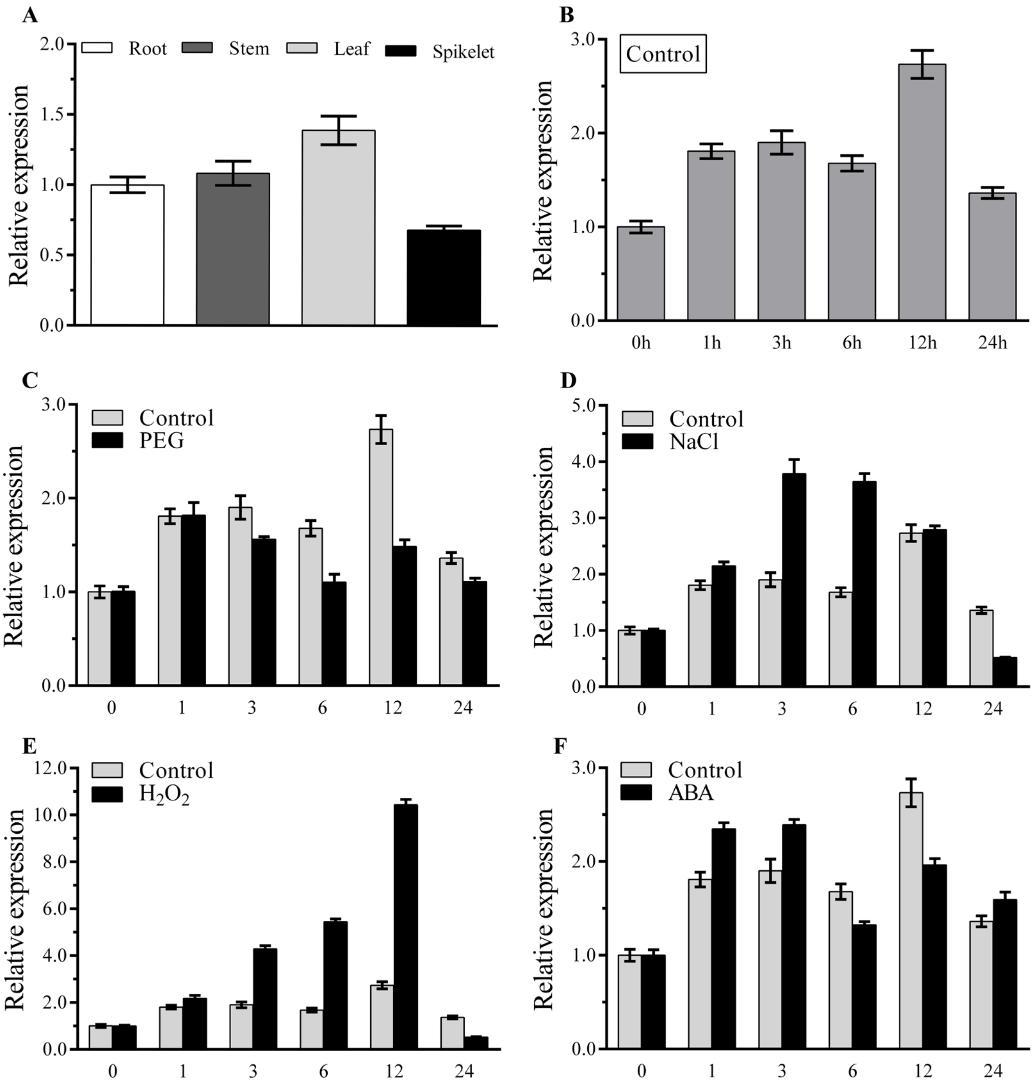

Figure S2. Expression patterns of BdGF14a in different organs and stress treatments in *B. distachyon* analysed by qRT-PCR analysis. (A) Different *B. distachyon* tissues including leaf , stem, root, and spikelet were sampled. The seedlings of *B. distachyon* were obtained under control (B), PEG (C), NaCl (D), H_2O_2 (E) and ABA (F) conditions. The relative expression of *BdGF14a* was analyzed by the $2^{-\Delta\Delta CT}$ method. Error bars are calculated from three replicates.

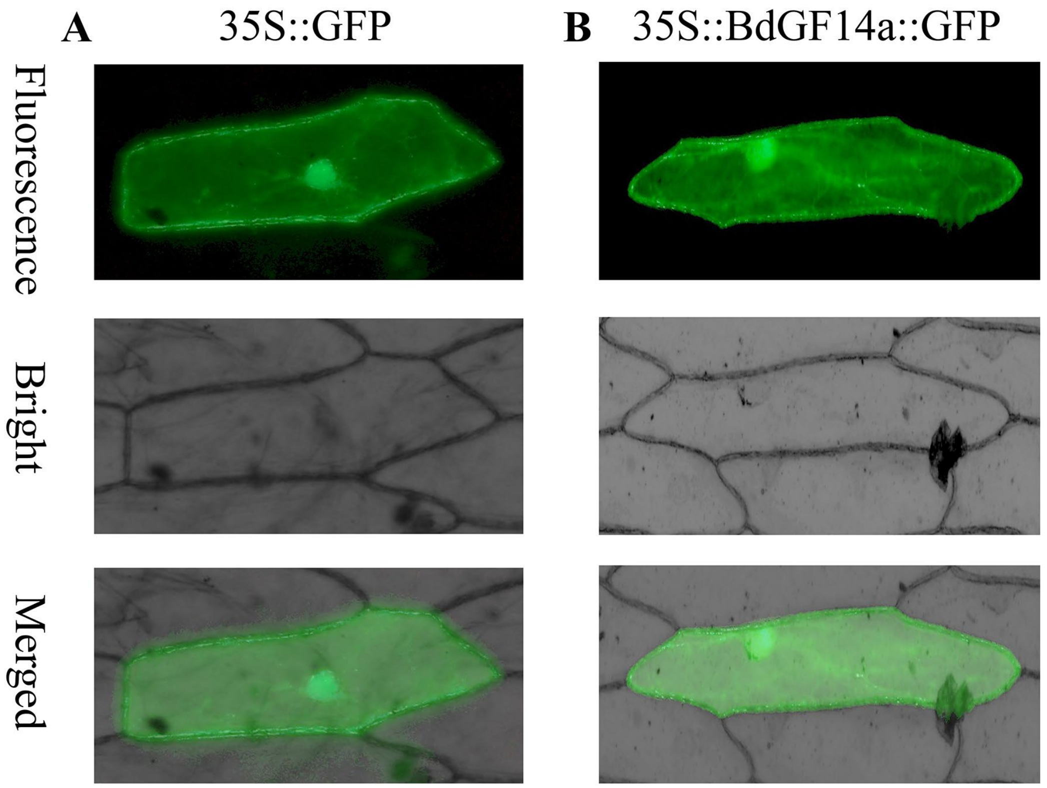

Figure S3. Subcellular localization of GFP and BdGF14a::GFP fusion protein in onion epidermal cells. The plasmids of *pBI121-BdGF14a-GFP* (B) and *PBI121-GFP* (control) (A) were introduced into onion epidermal cells by particle bombardment. The green fluorescence signals were observed by fluorescence microscopy (IX71, OLYMPUS, Japan).

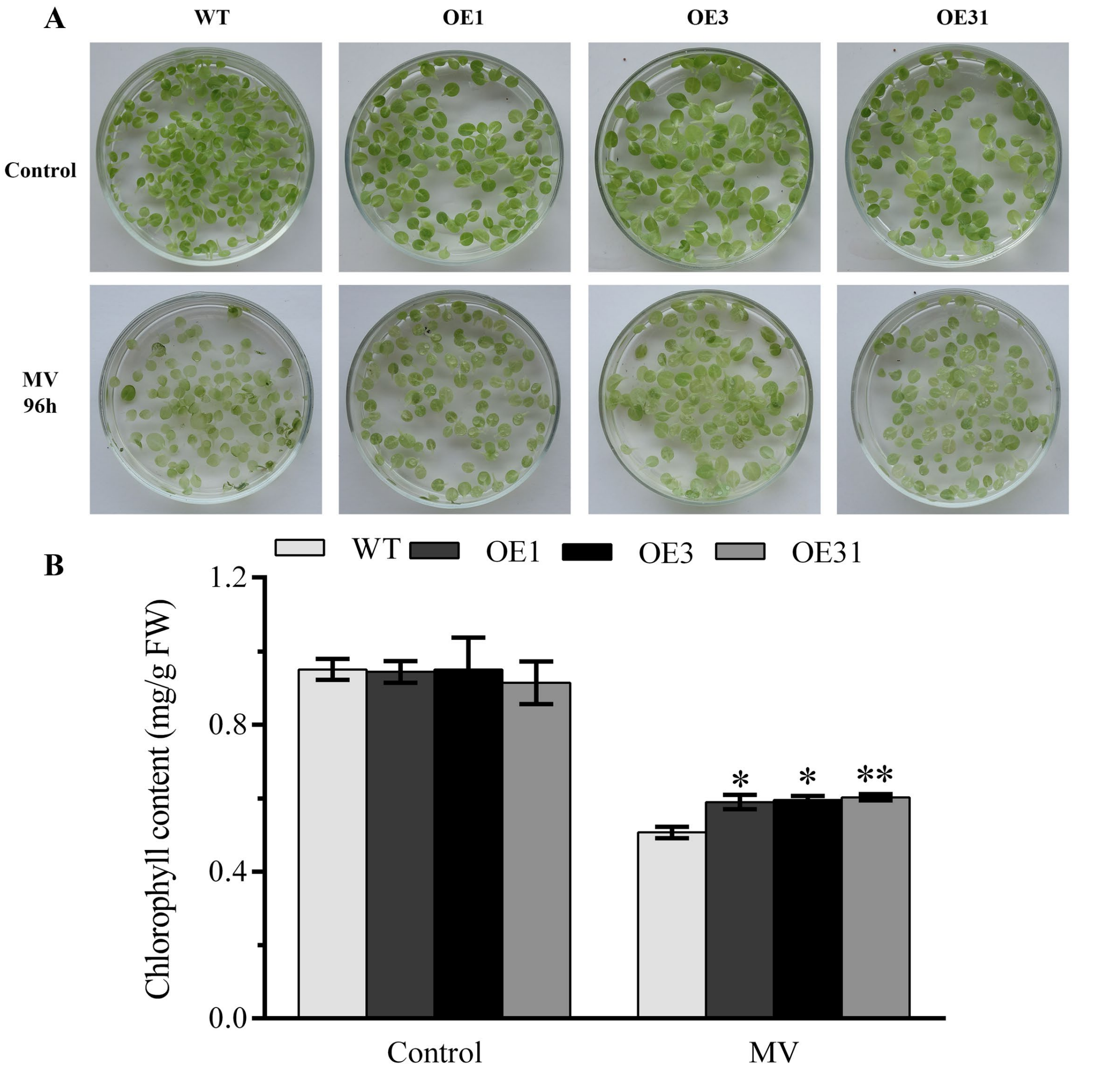

Figure S4. Oxidative tolerance analysis of *BdGF14a*-overexpressing and WT tobaccos under MV treatment. The leaves of 3-week-old *BdGF14a*-overexpressing and WT tobaccos were stained at distilled water with or without MV. The leaves were treated by MV for 96 h. (A) The photographs after treatment. (B) The chlorophyll content. Error bars are calculated from three replicates. Asterisks indicate statistically significant variations (**P* < 0.05; ***P* < 0.01).

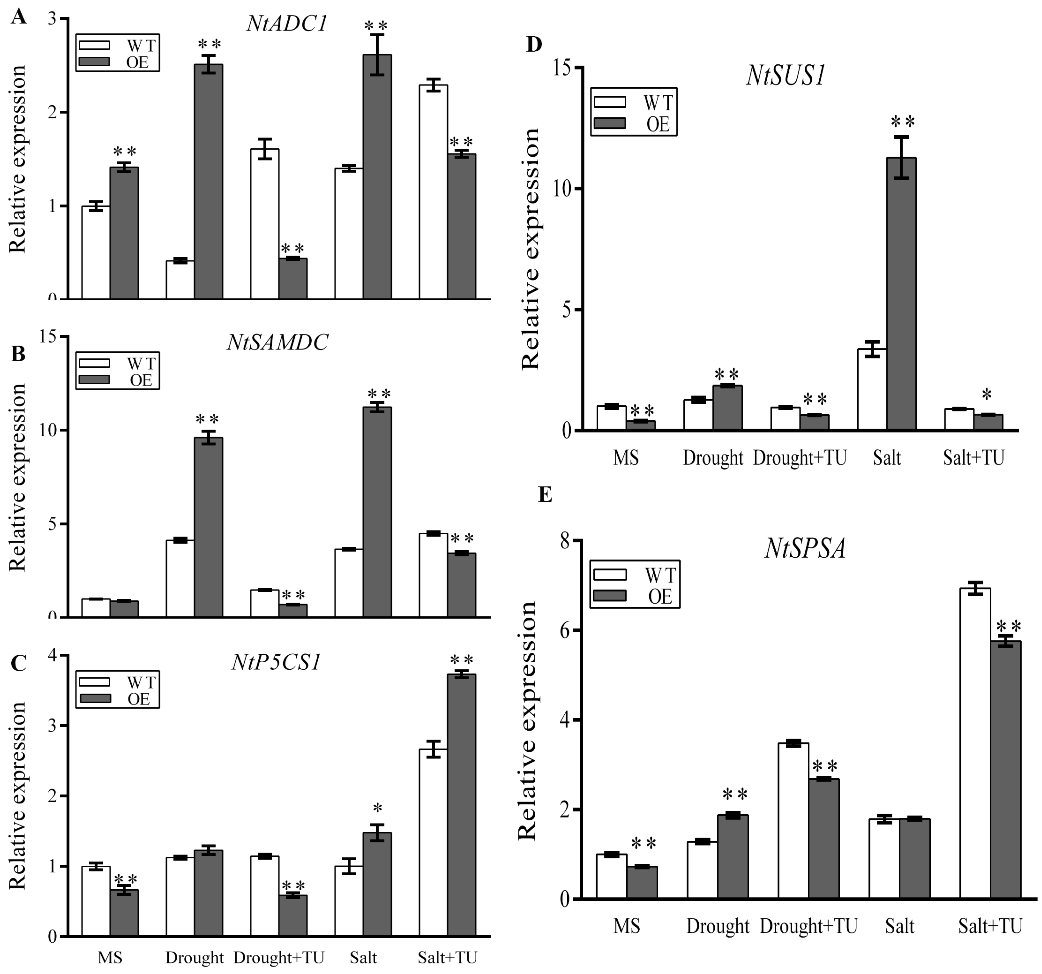

Figure S5. Expression patterns of osmolytes (polyamine, soluble sugar, and proline) biosynthesis-related genes in BdGF14a-transgenic tobaccos under stress-inducing conditions. Leaves of OE and WT Seedlings grew under normal, and stress treatments (drought and salt) with or without the sodium tungstate (Tu), were obtained and total RNAs were extracted. These genes including *NtADC1* (A), *NtSAMDC* (B), *NtP5CS1* (C), *NtSUS1* (D) and *NtSPSA* (E) were analyzed by qRT-PCR. Error bars are calculated from three replicates. Asterisks indicate statistically significant variations (**P* < 0.05; ***P* < 0.01).

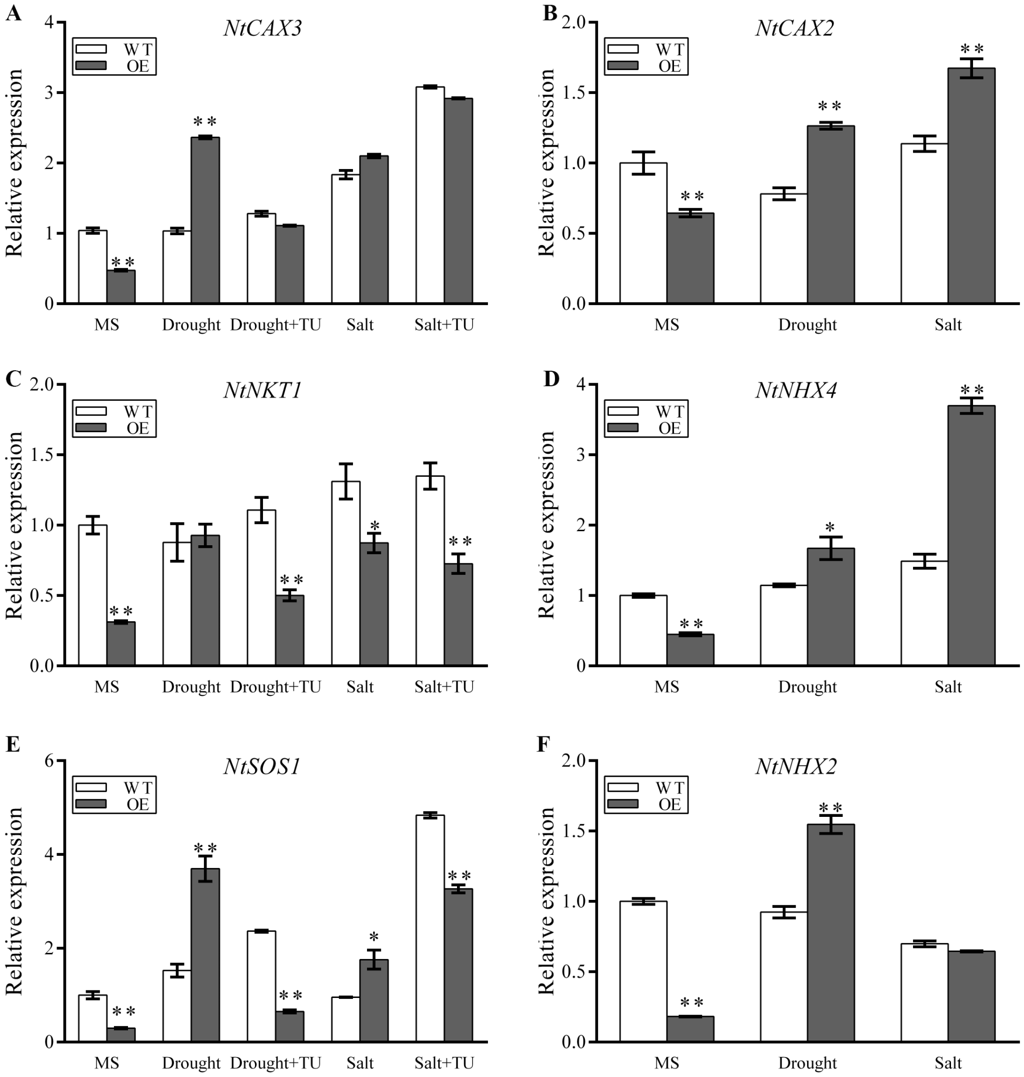

Figure S6. Expression patterns of ion channel-related genes in BdGF14a-transgenic tobaccos under under abiotic stresses. Leaves of WT and OE seedlings grew under normal, stress treatments (drought and salt) with or without Tu, were obtained and total RNAs were extracted. The ion channel related genes including *NtCAX3* (A), *NtCAX2* (B), *NtNKT1* (C), *NtNHX4* (D), *NtSOS1* (E) and *NtNHX2* (F) were analyzed by qRT-PCR. Error bars are calculated from three replicates. Asterisks indicate statistically apparent variations (**P* < 0.05; ***P* < 0.01).

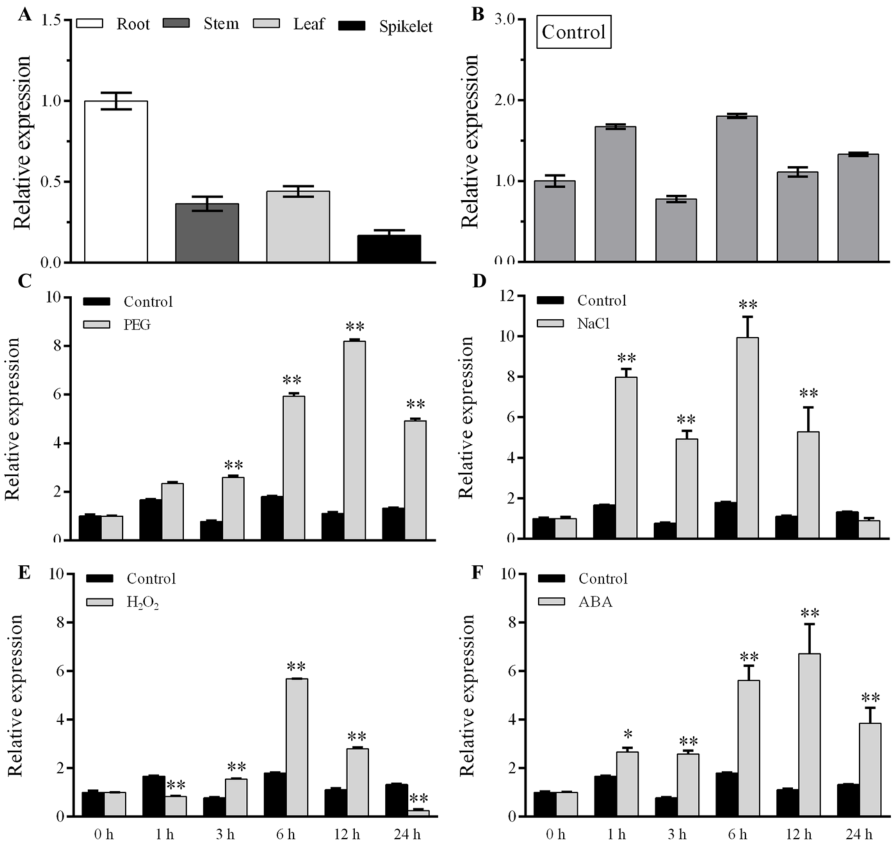

Figure S7. Expression patterns of BdbZIP62 in different organs and stress treatments by qRT-PCR analysis in B. distachyon

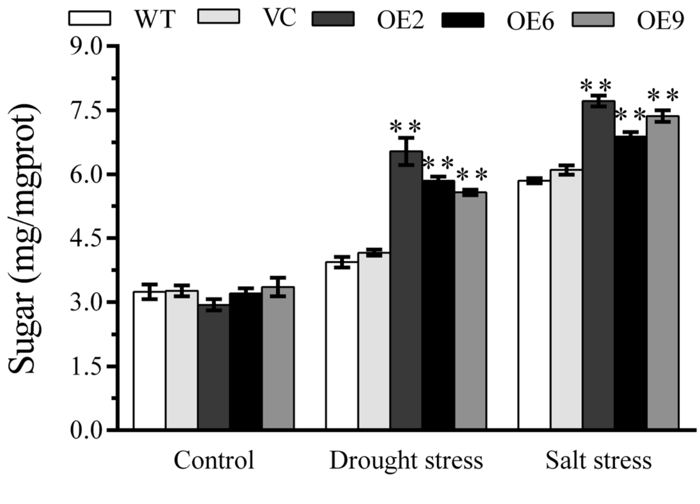

Figure S8. The sugar contents in BdbZIP62-transgenic tobaccos under salinity and drought. Error bars are calculated from three replicates. Asterisks indicate statistically apparent differences (**P* < 0.05; ***P* < 0.01).

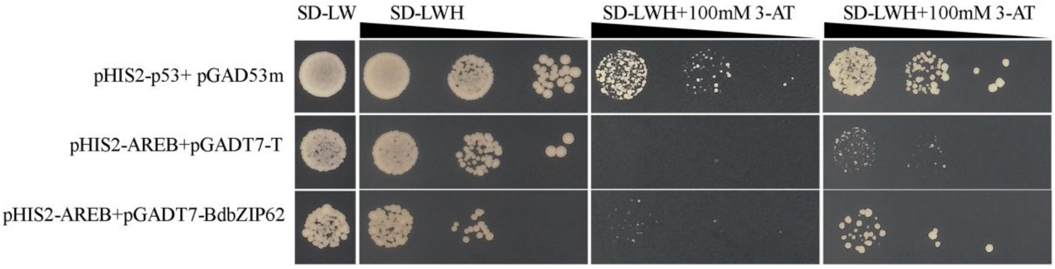

Figure S9. Y1H assay of BdbZIP62 interacted with ABRE *cis*-element. Y1H assay of BdbZIP62 interacted with ABRE *cis*-element. The recombinant vectors pHIS2-ABRE and pGADT7-BdbZIP62 were cotransformed into the Y187, and was plated on the SD-LW medium at 30°C for 2–3 d and then spotted onto SD-LW, SD-LWH, SD-LWH+100 mM 3-AT for another 3 d and 5 d. The cotransformed pHIS2-ABRE with pGADT7 empty vector, and pGAD53m with pHIS2-P53 were used as the negative and positive controls.
